# Carbon­yl{3,3′-di-*tert*-butyl-5,5′-dimeth­oxy-2,2′-bis­[(4,4,5,5-tetra­methyl-1,3,2-dioxaphospho­lan-2-yl)­oxy]biphenyl-κ^2^ *P*,*P*′}hydrido(triphenyl­phosphane-κ*P*)rhodium(I) diethyl ether tris­olvate

**DOI:** 10.1107/S1600536812049124

**Published:** 2012-12-05

**Authors:** Detlef Selent, Anke Spannenberg, Armin Börner

**Affiliations:** a Leibniz-Institut für Katalyse e. V. an der Universität Rostock, Albert-Einstein-Strasse 29a, 18059 Rostock, Germany

## Abstract

In the title compound, [RhH(C_74_H_68_O_8_P_2_)(C_18_H_15_P)(CO)]·3C_4_H_10_O, the CHP_3_ coordination set at the Rh^I^ ion is arranged in a distorted trigonal–bipyramidal geometry with the P atoms adopting equatorial coordination sites and the C atom of the carbonyl ligand as well as the H atom adopting the axial sites. The asymmetric unit contains two very similar mol­ecules of the rhodium complex, two half-occupied diethyl ether mol­ecules and further diethyl ether solvent mol­ecules which could not be modelled successfully. Therefore contributions of the latter were removed from the diffraction data using the SQUEEZE procedure in *PLATON* [Spek (2009 ▶). *Acta Cryst.* D**65**, 148–155].

## Related literature

For the solid-state structure and for DFT calculations of the dicarbonyl precursor of the title compound, see: Selent *et al.* (2011 ▶, 2012 ▶). For the crystal structure of another diphosphite hydrido complex of rhodium(I), see: van Rooy *et al.* (1995 ▶, 1996 ▶). An octa­hedral Rh^III^ hydrido complex with both diphosphite and triphenyl­phosphane ligands adopting coordination sites in the same plane has been characterized structurally, see: Rubio *et al.* (2009 ▶).
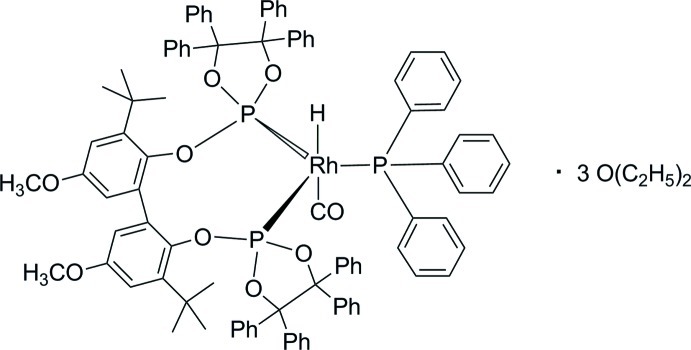


## Experimental

### 

#### Crystal data

[RhH(C_74_H_68_O_8_P_2_)(C_18_H_15_P)(CO)]·3C_4_H_10_O
*M* *_r_* = 1763.78
Triclinic, 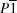

*a* = 21.1611 (4) Å
*b* = 22.1298 (4) Å
*c* = 22.6020 (4) Å
α = 112.345 (1)°
β = 101.135 (1)°
γ = 96.757 (1)°
*V* = 9389.9 (3) Å^3^
*Z* = 4
Mo *K*α radiation
μ = 0.29 mm^−1^
*T* = 150 K
0.44 × 0.39 × 0.18 mm

#### Data collection

Bruker Kappa APEXII DUO diffractometer
Absorption correction: multi-scan (*SADABS*; Bruker, 2008 ▶) *T*_min_ = 0.94, *T*_max_ = 1.00
388867 measured reflections
43155 independent reflections
32017 reflections with *I* > 2σ(*I*)
*R*_int_ = 0.073

#### Refinement

*R*[*F* ^2^ > 2σ(*F* ^2^)] = 0.053
*wR*(*F* ^2^) = 0.152
*S* = 1.05
43155 reflections
1866 parameters
55 restraints
H atoms treated by a mixture of independent and constrained refinement
Δρ_max_ = 1.43 e Å^−3^
Δρ_min_ = −0.99 e Å^−3^

### 

Data collection: *APEX2* (Bruker, 2011 ▶); cell refinement: *SAINT* (Bruker, 2009 ▶); data reduction: *SAINT*; program(s) used to solve structure: *SHELXS97* (Sheldrick, 2008 ▶); program(s) used to refine structure: *SHELXL97* (Sheldrick, 2008 ▶); molecular graphics: *XP* in *SHELXTL* (Sheldrick, 2008 ▶); software used to prepare material for publication: *SHELXTL*.

## Supplementary Material

Crystal structure: contains datablock(s) I, global. DOI: 10.1107/S1600536812049124/wm2697sup1.cif

Structure factors: contains datablock(s) I. DOI: 10.1107/S1600536812049124/wm2697Isup2.hkl

Additional supplementary materials:  crystallographic information; 3D view; checkCIF report

## Figures and Tables

**Fig. 1.**
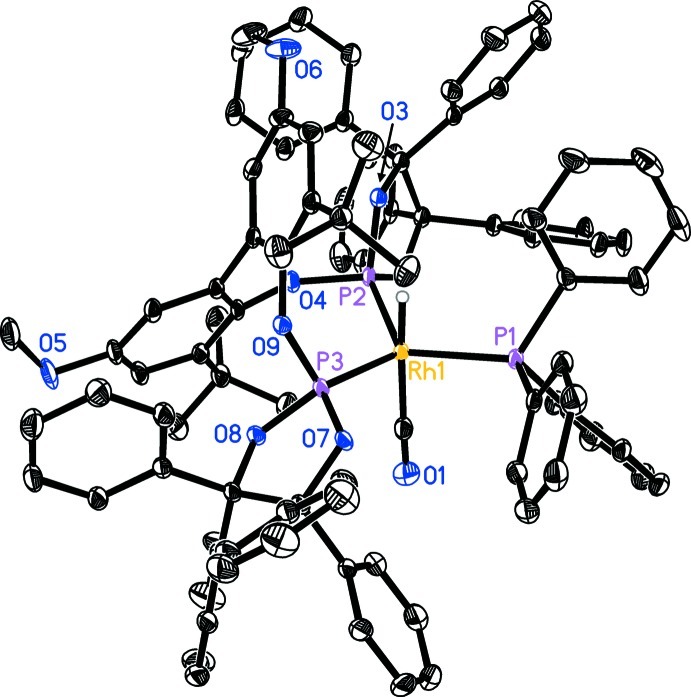
The molecular structure of the title compound. Only one of the two complex molecules in the asymmetric unit is depicted. Hydrogen atoms (except the hydride ligand) and the diethyl ether molecules were omitted for clarity. Displacement ellipsoids are drawn at the 30% probability level.

## supplementary crystallographic information

### Comment

The title compound, [Rh(C_74_H_68_O_8_P_2_)(C_18_H_15_P)H(CO)]^.^3C_4_H_10_O, is formed by reaction of {3,3'-di-*tert*-butyl-5,5'- dimethoxy-2,2'-bis[(1,1,2,2-tetraphenyl-1,2-ethandioxy)phosphanyloxy- κ*P*]biphenyl}-dicarbonylhydridorhodium(I) diethyl ether solvate with triphenylphosphane in toluene which affords substitution of one carbonyl ligand. The substitution reaction induces the rearrangement of the diphosphite ligand resulting in a bis-equatorial coordination.

The asymmetric unit of the title compound contains two molecules of the rhodium complex, two half-occupied diethyl ether molecules and further solvent molecules (diethyl ether) which are partly occupied and highly disordered. The latter could not be modelled successfully. Therefore, contributions of them were removed from the diffraction data with the SQUEEZE procedure in *PLATON* (Spek, 2009).

The distances Rh1—P2 = 2.2447 (7), Rh1—P3 = 2.2569 (7), Rh2—P5 = 2.2555 (7) and Rh2—P6 = 2.2553 (7) Å do significantly differ from that of the precursor complex (Rh1—P1 = 2.3045 (5), Rh1—P2 = 2.2913 (5) Å), as do the angles P2—Rh1—P3 = 117.18 (2) and P5—Rh2—P6 = 117.99 (2)° (P1—Rh1—P2 = 109.66 (2)°, Selent *et al.*, 2012). The latter, together with the P—Rh—P angles arising from combinations with the triphenylphosphane phosphorus atoms, approach the ideal value of a trigonal-planar arrangement. The hydride ligands could be found from the difference Fourier map. The Rh—H distances were refined to 1.40 (4) Å.

Related rhodium complexes have been described by Rubio *et al.* (2009); Selent *et al.* (2011, 2012) and van Rooy *et al.* (1995, 1996).

### Experimental

To a stirred solution of {3,3'-di-*tert*-butyl-5,5'-dimethoxy-2,2'- bis[(1,1,2,2-tetraphenyl-1,2-ethandioxy)phosphanyloxy-κ*P*]biphenyl}- dicarbonylhydridorhodium(I) diethyl ether solvate (0.1755 g, 0.134 mmol) in toluene (2 ml) a solution of triphenylphosphane (0.035 g, 0.134 mmol) in toluene (2 ml) was added dropwise at room temperature. The resulting yellow solution was stirred for 20 h at 343 K, then filtered and the filtrate evaporated to dryness. Recrystallization of the crude product from a mixture of toluene and diethyl ether at 278 K afforded colorless crystals which were filtered off and dried *in vacuo* at room temperature for 1 h. Yield: 120 mg (0.076 mmol, 56%).

### Refinement

The hydride ligands could be found from a difference Fourier map and were refined freely. All other H atoms were placed in idealized positions with *d*(C—H) = 0.95 Å (CH), 0.99 Å (CH_2_) and 0.98 Å (CH_3_) and refined using a riding model with *U*_iso_(H) fixed at 1.2*U*_eq_(C) for CH, CH_2_ and 1.5*U*_eq_(C) for CH_3_. Bond lengths and angles of several phenyl rings were constrained to idealized values with C—C distances of 1.39 Å. DFIX and SADI instructions in SHELXL (Sheldrick, 2008) were used to improve the geometry of the half-occupied diethyl ether molecules. Additionally, anisotropic displacement parameters of C atoms of one solvent molecule were restrained to be equal using the SIMU instruction. This was also necessary for those of one phenyl ring (C181—C186) due to unresolved disorder. Contributions of further disordered diethyl ether solvent molecules were removed from the diffraction data with *PLATON* using the SQUEEZE procedure (Spek, 2009). SQUEEZE estimated the electron count in the void volume of 1790 Å^3^ to be 213 which is in good agreement with two and a half further diethyl ether molecules present in the crystal, leading to an overall solvent number of three per complex molecule.

### Crystal data

**Table:**

[RhH(C_74_H_68_O_8_P_2_)(C_18_H_15_P)(CO)]·3C_4_H_10_O	*Z* = 4
*M**_r_* = 1763.78	*F*(000) = 3720
Triclinic, *P*1	*D*_x_ = 1.248 Mg m^−^^3^
*a* = 21.1611 (4) Å	Mo *K*α radiation, λ = 0.71073 Å
*b* = 22.1298 (4) Å	Cell parameters from 9259 reflections
*c* = 22.6020 (4) Å	θ = 2.3–28.7°
α = 112.345 (1)°	µ = 0.29 mm^−^^1^
β = 101.135 (1)°	*T* = 150 K
γ = 96.757 (1)°	Prism, colorless
*V* = 9389.9 (3) Å^3^	0.44 × 0.39 × 0.18 mm

### Data collection

**Table:**

Bruker Kappa APEXII DUO diffractometer	43155 independent reflections
Radiation source: fine-focus sealed tube	32017 reflections with *I* > 2σ(*I*)
Curved graphite monochromator	*R*_int_ = 0.073
Detector resolution: 8.3333 pixels mm^-1^	θ_max_ = 27.5°, θ_min_ = 1.5°
ω scans	*h* = −27→27
Absorption correction: multi-scan (*SADABS*; Bruker, 2008)	*k* = −28→28
*T*_min_ = 0.94, *T*_max_ = 1.00	*l* = −29→29
388867 measured reflections	

### Refinement

**Table:**

Refinement on *F*^2^	Primary atom site location: structure-invariant direct methods
Least-squares matrix: full	Secondary atom site location: difference Fourier map
*R*[*F*^2^ > 2σ(*F*^2^)] = 0.053	Hydrogen site location: inferred from neighbouring sites
*wR*(*F*^2^) = 0.152	H atoms treated by a mixture of independent and constrained refinement
*S* = 1.05	*w* = 1/[σ^2^(*F*_o_^2^) + (0.0834*P*)^2^ + 5.5129*P*] where *P* = (*F*_o_^2^ + 2*F*_c_^2^)/3
43155 reflections	(Δ/σ)_max_ = 0.001
1866 parameters	Δρ_max_ = 1.43 e Å^−^^3^
55 restraints	Δρ_min_ = −0.99 e Å^−^^3^

### Special details

**Table:**

Geometry. All e.s.d.'s (except the e.s.d. in the dihedral angle between two l.s. planes) are estimated using the full covariance matrix. The cell e.s.d.'s are taken into account individually in the estimation of e.s.d.'s in distances, angles and torsion angles; correlations between e.s.d.'s in cell parameters are only used when they are defined by crystal symmetry. An approximate (isotropic) treatment of cell e.s.d.'s is used for estimating e.s.d.'s involving l.s. planes.
Refinement. Refinement of *F*^2^ against ALL reflections. The weighted *R*-factor *wR* and goodness of fit *S* are based on *F*^2^, conventional *R*-factors *R* are based on *F*, with *F* set to zero for negative *F*^2^. The threshold expression of *F*^2^ > σ(*F*^2^) is used only for calculating *R*-factors(gt) *etc*. and is not relevant to the choice of reflections for refinement. *R*-factors based on *F*^2^ are statistically about twice as large as those based on *F*, and *R*- factors based on ALL data will be even larger.

### Fractional atomic coordinates and isotropic or equivalent isotropic displacement parameters (Å^2^)

**Table:**

	*x*	*y*	*z*	*U*_iso_*/*U*_eq_	Occ. (<1)
C181	−0.14393 (10)	0.43500 (17)	0.21835 (17)	0.0714 (8)	
C182	−0.18041 (12)	0.46717 (16)	0.18632 (17)	0.0725 (9)	
H182	−0.1587	0.4978	0.1728	0.087*	
C183	−0.24866 (12)	0.45451 (19)	0.17406 (18)	0.0876 (10)	
H183	−0.2736	0.4765	0.1522	0.105*	
C184	−0.28042 (10)	0.40967 (19)	0.1938 (2)	0.1025 (11)	
H184	−0.3271	0.4010	0.1855	0.123*	
C185	−0.24395 (13)	0.37750 (18)	0.2259 (2)	0.1012 (10)	
H185	−0.2657	0.3469	0.2394	0.121*	
C186	−0.17570 (13)	0.39016 (18)	0.23812 (19)	0.0873 (9)	
H186	−0.1508	0.3682	0.2600	0.105*	
O19	0.1836 (3)	0.8334 (3)	0.1238 (3)	0.0631 (15)	0.50
C187	0.1235 (3)	0.8479 (4)	0.0984 (4)	0.077 (2)	0.50
H18A	0.1114	0.8838	0.1338	0.093*	0.50
H18B	0.0875	0.8077	0.0794	0.093*	0.50
C188	0.1356 (4)	0.8703 (4)	0.0452 (4)	0.070 (2)	0.50
H18C	0.0955	0.8815	0.0258	0.105*	0.50
H18D	0.1474	0.8342	0.0106	0.105*	0.50
H18E	0.1717	0.9098	0.0648	0.105*	0.50
C189	0.1735 (5)	0.8107 (6)	0.1728 (5)	0.098 (3)	0.50
H18F	0.1392	0.7690	0.1534	0.117*	0.50
H18G	0.1605	0.8451	0.2088	0.117*	0.50
C190	0.2406 (5)	0.7985 (6)	0.1977 (6)	0.102 (3)	0.50
H19A	0.2391	0.7824	0.2323	0.154*	0.50
H19B	0.2736	0.8403	0.2159	0.154*	0.50
H19C	0.2525	0.7649	0.1609	0.154*	0.50
C1	0.57231 (16)	0.83998 (16)	0.26418 (15)	0.0344 (7)	
C2	0.63930 (9)	0.99152 (8)	0.45993 (9)	0.0312 (6)	
C3	0.57380 (8)	0.99575 (9)	0.44256 (8)	0.0379 (7)	
H3	0.5431	0.9593	0.4069	0.046*	
C4	0.55321 (8)	1.05328 (11)	0.47742 (10)	0.0463 (9)	
H4	0.5084	1.0562	0.4655	0.056*	
C5	0.59811 (11)	1.10659 (9)	0.52964 (10)	0.0481 (9)	
H5	0.5840	1.1459	0.5535	0.058*	
C6	0.66360 (9)	1.10236 (9)	0.54702 (9)	0.0461 (8)	
H6	0.6943	1.1388	0.5827	0.055*	
C7	0.68420 (7)	1.04483 (10)	0.51216 (9)	0.0368 (7)	
H7	0.7290	1.0419	0.5240	0.044*	
C8	0.61093 (9)	0.84922 (9)	0.41973 (10)	0.0330 (7)	
C9	0.57987 (11)	0.86408 (9)	0.47088 (9)	0.0393 (8)	
H9	0.5848	0.9092	0.5010	0.047*	
C10	0.54166 (11)	0.81294 (12)	0.47794 (10)	0.0499 (9)	
H10	0.5204	0.8231	0.5129	0.060*	
C11	0.53451 (11)	0.74694 (10)	0.43386 (12)	0.0552 (10)	
H11	0.5084	0.7120	0.4387	0.066*	
C12	0.56557 (12)	0.73208 (8)	0.38271 (11)	0.0543 (9)	
H12	0.5607	0.6870	0.3526	0.065*	
C13	0.60378 (11)	0.78322 (10)	0.37564 (9)	0.0408 (8)	
H13	0.6250	0.7731	0.3407	0.049*	
C14	0.74426 (8)	0.92469 (10)	0.46391 (8)	0.0321 (6)	
C15	0.74770 (9)	0.91243 (11)	0.52025 (10)	0.0421 (8)	
H15	0.7094	0.8906	0.5260	0.050*	
C16	0.80712 (12)	0.93219 (12)	0.56824 (8)	0.0535 (10)	
H16	0.8095	0.9238	0.6067	0.064*	
C17	0.86311 (9)	0.96420 (12)	0.55989 (10)	0.0560 (11)	
H17	0.9037	0.9777	0.5927	0.067*	
C18	0.85968 (8)	0.97646 (12)	0.50354 (11)	0.0509 (9)	
H18	0.8979	0.9983	0.4978	0.061*	
C19	0.80026 (9)	0.95671 (11)	0.45556 (9)	0.0402 (8)	
H19	0.7979	0.9651	0.4171	0.048*	
C20	0.76745 (14)	0.73280 (14)	0.23918 (14)	0.0277 (6)	
C21	0.82714 (14)	0.79459 (13)	0.25618 (13)	0.0253 (6)	
C22	0.76466 (14)	0.71297 (15)	0.29712 (14)	0.0294 (6)	
C23	0.76259 (15)	0.76163 (15)	0.35759 (14)	0.0314 (6)	
H23	0.7636	0.8063	0.3625	0.038*	
C24	0.75911 (16)	0.74548 (17)	0.41073 (15)	0.0371 (7)	
H24	0.7586	0.7793	0.4519	0.045*	
C25	0.75643 (18)	0.68015 (17)	0.40378 (16)	0.0423 (8)	
H25	0.7540	0.6690	0.4400	0.051*	
C26	0.7573 (2)	0.63168 (18)	0.34410 (17)	0.0474 (9)	
H26	0.7549	0.5867	0.3389	0.057*	
C27	0.76189 (18)	0.64815 (16)	0.29107 (16)	0.0415 (8)	
H27	0.7631	0.6143	0.2502	0.050*	
C28	0.76360 (10)	0.67045 (8)	0.17628 (8)	0.0317 (6)	
C29	0.70394 (8)	0.63975 (10)	0.12864 (10)	0.0414 (8)	
H29	0.6662	0.6585	0.1339	0.050*	
C30	0.69953 (10)	0.58161 (10)	0.07329 (9)	0.0539 (10)	
H30	0.6588	0.5606	0.0407	0.065*	
C31	0.75479 (12)	0.55416 (9)	0.06559 (9)	0.0518 (10)	
H31	0.7518	0.5144	0.0278	0.062*	
C32	0.81446 (10)	0.58486 (10)	0.11324 (10)	0.0435 (8)	
H32	0.8522	0.5661	0.1080	0.052*	
C33	0.81887 (8)	0.64300 (9)	0.16858 (9)	0.0368 (7)	
H33	0.8596	0.6640	0.2011	0.044*	
C34	0.86246 (14)	0.78453 (14)	0.20024 (14)	0.0289 (6)	
C35	0.82838 (16)	0.77862 (15)	0.13847 (14)	0.0326 (6)	
H35	0.7827	0.7792	0.1302	0.039*	
C36	0.86027 (18)	0.77191 (16)	0.08858 (16)	0.0403 (7)	
H36	0.8364	0.7685	0.0469	0.048*	
C37	0.92653 (18)	0.77015 (17)	0.09948 (17)	0.0445 (8)	
H37	0.9484	0.7664	0.0657	0.053*	
C38	0.96053 (18)	0.77392 (17)	0.15953 (18)	0.0439 (8)	
H38	1.0057	0.7713	0.1668	0.053*	
C39	0.92890 (16)	0.78156 (17)	0.20955 (17)	0.0398 (8)	
H39	0.9531	0.7848	0.2511	0.048*	
C40	0.88030 (14)	0.81697 (15)	0.32105 (14)	0.0281 (6)	
C41	0.90873 (15)	0.77224 (17)	0.34205 (15)	0.0349 (7)	
H41	0.8907	0.7257	0.3190	0.042*	
C42	0.96277 (17)	0.7950 (2)	0.39597 (18)	0.0474 (9)	
H42	0.9812	0.7640	0.4099	0.057*	
C43	0.99033 (17)	0.8625 (2)	0.42983 (18)	0.0501 (9)	
H43	1.0277	0.8779	0.4668	0.060*	
C44	0.96293 (17)	0.90744 (19)	0.40935 (17)	0.0453 (8)	
H44	0.9816	0.9539	0.4321	0.054*	
C45	0.90819 (15)	0.88456 (16)	0.35559 (15)	0.0351 (7)	
H45	0.8895	0.9158	0.3422	0.042*	
C46	0.67384 (14)	0.86482 (15)	0.13299 (13)	0.0273 (6)	
C47	0.62165 (14)	0.83177 (16)	0.07520 (14)	0.0317 (6)	
C48	0.60251 (16)	0.86930 (17)	0.03994 (14)	0.0353 (7)	
H48	0.5679	0.8485	0.0004	0.042*	
C49	0.63162 (16)	0.93487 (17)	0.06013 (14)	0.0370 (7)	
C50	0.68016 (15)	0.96774 (15)	0.11859 (14)	0.0307 (6)	
H50	0.6986	1.0139	0.1337	0.037*	
C51	0.70198 (14)	0.93198 (14)	0.15555 (13)	0.0265 (6)	
C52	0.75892 (14)	0.97027 (14)	0.21497 (13)	0.0256 (6)	
C53	0.82131 (14)	0.95697 (14)	0.21089 (14)	0.0279 (6)	
H53	0.8264	0.9215	0.1731	0.033*	
C54	0.87523 (14)	0.99607 (15)	0.26254 (16)	0.0320 (6)	
C55	0.86843 (15)	1.04907 (15)	0.31751 (16)	0.0327 (6)	
H55	0.9065	1.0753	0.3523	0.039*	
C56	0.80727 (14)	1.06460 (14)	0.32281 (14)	0.0279 (6)	
C57	0.75257 (13)	1.02281 (14)	0.27075 (13)	0.0255 (6)	
C58	0.58923 (16)	0.75755 (17)	0.04770 (15)	0.0382 (7)	
C59	0.52858 (19)	0.7380 (2)	−0.01132 (18)	0.0547 (10)	
H59A	0.5068	0.6914	−0.0252	0.082*	
H59B	0.5427	0.7431	−0.0483	0.082*	
H59C	0.4977	0.7671	0.0020	0.082*	
C60	0.63865 (19)	0.71500 (18)	0.02257 (17)	0.0469 (8)	
H60A	0.6778	0.7261	0.0590	0.070*	
H60B	0.6514	0.7242	−0.0131	0.070*	
H60C	0.6183	0.6675	0.0058	0.070*	
C61	0.56379 (18)	0.74027 (19)	0.09960 (17)	0.0456 (8)	
H61A	0.6010	0.7479	0.1368	0.068*	
H61B	0.5410	0.6932	0.0796	0.068*	
H61C	0.5331	0.7686	0.1155	0.068*	
C62	0.6526 (2)	1.0214 (2)	0.0235 (2)	0.0568 (10)	
H62A	0.6602	1.0573	0.0678	0.085*	
H62B	0.6328	1.0364	−0.0100	0.085*	
H62C	0.6946	1.0098	0.0165	0.085*	
C63	0.94820 (18)	0.93748 (18)	0.2054 (2)	0.0538 (10)	
H63A	0.9232	0.8939	0.1979	0.081*	
H63B	0.9951	0.9363	0.2110	0.081*	
H63C	0.9324	0.9478	0.1673	0.081*	
C64	0.80299 (15)	1.12667 (15)	0.38167 (14)	0.0311 (6)	
C65	0.87094 (17)	1.16401 (18)	0.42862 (17)	0.0472 (8)	
H65A	0.8665	1.2042	0.4646	0.071*	
H65B	0.8995	1.1769	0.4042	0.071*	
H65C	0.8904	1.1349	0.4471	0.071*	
C66	0.77412 (19)	1.17556 (17)	0.35638 (17)	0.0448 (8)	
H66A	0.7288	1.1548	0.3292	0.067*	
H66B	0.8009	1.1866	0.3296	0.067*	
H66C	0.7742	1.2165	0.3942	0.067*	
C67	0.76009 (18)	1.10851 (18)	0.42264 (16)	0.0447 (8)	
H67A	0.7159	1.0848	0.3941	0.067*	
H67B	0.7570	1.1494	0.4586	0.067*	
H67C	0.7800	1.0797	0.4413	0.067*	
C68	0.53496 (14)	1.05328 (16)	0.28807 (14)	0.0303 (6)	
C69	0.53080 (14)	1.01804 (15)	0.20919 (13)	0.0285 (6)	
C70	0.54424 (10)	1.12928 (8)	0.32133 (9)	0.0346 (7)	
C71	0.58078 (11)	1.16100 (10)	0.38704 (9)	0.0447 (8)	
H71	0.6034	1.1365	0.4074	0.054*	
C72	0.58424 (12)	1.22864 (11)	0.42292 (8)	0.0572 (10)	
H72	0.6092	1.2503	0.4678	0.069*	
C73	0.55115 (13)	1.26456 (8)	0.39311 (11)	0.0585 (10)	
H73	0.5535	1.3108	0.4176	0.070*	
C74	0.51461 (12)	1.23284 (10)	0.32741 (11)	0.0494 (9)	
H74	0.4920	1.2574	0.3070	0.059*	
C75	0.51116 (10)	1.16520 (10)	0.29152 (8)	0.0396 (7)	
H75	0.4862	1.1435	0.2466	0.048*	
C76	0.47551 (8)	1.02122 (11)	0.30545 (10)	0.0369 (7)	
C77	0.43269 (11)	1.05876 (10)	0.33438 (10)	0.0470 (9)	
H77	0.4390	1.1050	0.3435	0.056*	
C78	0.38070 (10)	1.02872 (14)	0.34997 (11)	0.0610 (12)	
H78	0.3514	1.0544	0.3697	0.073*	
C79	0.37154 (9)	0.96113 (15)	0.33662 (13)	0.0712 (14)	
H79	0.3360	0.9406	0.3473	0.085*	
C80	0.41436 (12)	0.92358 (11)	0.30769 (13)	0.0625 (12)	
H80	0.4081	0.8774	0.2986	0.075*	
C81	0.46635 (10)	0.95362 (11)	0.29210 (11)	0.0447 (8)	
H81	0.4956	0.9280	0.2723	0.054*	
C82	0.55575 (9)	1.06648 (9)	0.17980 (9)	0.0321 (7)	
C83	0.61258 (9)	1.11574 (10)	0.21531 (8)	0.0381 (7)	
H83	0.6367	1.1188	0.2568	0.046*	
C84	0.63407 (9)	1.16046 (10)	0.19014 (11)	0.0488 (9)	
H84	0.6729	1.1941	0.2144	0.059*	
C85	0.59872 (12)	1.15592 (12)	0.12945 (12)	0.0602 (11)	
H85	0.6134	1.1865	0.1122	0.072*	
C86	0.54189 (11)	1.10666 (13)	0.09394 (9)	0.0538 (10)	
H86	0.5177	1.1036	0.0525	0.065*	
C87	0.52041 (9)	1.06194 (10)	0.11912 (9)	0.0403 (8)	
H87	0.4816	1.0283	0.0948	0.048*	
C88	0.46316 (15)	0.97476 (17)	0.16744 (14)	0.0337 (7)	
C89	0.40626 (15)	1.00146 (18)	0.16901 (15)	0.0378 (7)	
H89	0.4098	1.0474	0.1958	0.045*	
C90	0.34496 (17)	0.9614 (2)	0.13177 (17)	0.0488 (9)	
H90	0.3068	0.9800	0.1335	0.059*	
C91	0.33892 (19)	0.8947 (2)	0.0922 (2)	0.0615 (11)	
H91	0.2967	0.8669	0.0678	0.074*	
C92	0.3949 (2)	0.8688 (2)	0.0885 (2)	0.0674 (12)	
H92	0.3914	0.8235	0.0599	0.081*	
C93	0.45604 (17)	0.90829 (19)	0.12602 (18)	0.0495 (9)	
H93	0.4940	0.8894	0.1233	0.059*	
C94	0.14778 (17)	0.42773 (16)	0.31172 (15)	0.0359 (7)	
C95	0.07871 (9)	0.25826 (7)	0.38265 (9)	0.0282 (6)	
C96	0.09739 (9)	0.25617 (8)	0.44416 (7)	0.0358 (7)	
H96	0.1217	0.2953	0.4817	0.043*	
C97	0.08046 (11)	0.19684 (10)	0.45073 (8)	0.0436 (8)	
H97	0.0932	0.1954	0.4928	0.052*	
C98	0.04483 (11)	0.13962 (8)	0.39579 (11)	0.0454 (9)	
H98	0.0333	0.0991	0.4003	0.055*	
C99	0.02615 (10)	0.14171 (7)	0.33428 (9)	0.0413 (8)	
H99	0.0018	0.1026	0.2967	0.050*	
C100	0.04309 (10)	0.20103 (9)	0.32772 (7)	0.0341 (7)	
H100	0.0303	0.2025	0.2857	0.041*	
C101	0.01423 (15)	0.36582 (15)	0.40033 (13)	0.0298 (6)	
C102	−0.03515 (15)	0.32549 (15)	0.40886 (15)	0.0338 (7)	
H102	−0.0310	0.2818	0.4044	0.041*	
C103	−0.09081 (17)	0.34881 (17)	0.42397 (17)	0.0425 (8)	
H103	−0.1246	0.3208	0.4296	0.051*	
C104	−0.09737 (17)	0.41216 (17)	0.43088 (17)	0.0429 (8)	
H104	−0.1355	0.4279	0.4413	0.052*	
C105	−0.04833 (18)	0.45252 (17)	0.42263 (17)	0.0438 (8)	
H105	−0.0526	0.4962	0.4273	0.053*	
C106	0.00737 (17)	0.42973 (16)	0.40760 (15)	0.0364 (7)	
H106	0.0411	0.4580	0.4022	0.044*	
C107	0.15474 (16)	0.39268 (15)	0.44692 (14)	0.0339 (7)	
C108	0.21837 (18)	0.39996 (18)	0.43974 (17)	0.0465 (8)	
H108	0.2259	0.3786	0.3973	0.056*	
C109	0.2713 (2)	0.4382 (2)	0.4940 (2)	0.0656 (12)	
H109	0.3149	0.4422	0.4889	0.079*	
C110	0.2598 (3)	0.4703 (2)	0.5555 (2)	0.0666 (13)	
H110	0.2957	0.4971	0.5926	0.080*	
C111	0.1968 (2)	0.46388 (18)	0.56333 (17)	0.0545 (11)	
H111	0.1894	0.4862	0.6057	0.065*	
C112	0.14446 (19)	0.42505 (16)	0.50976 (15)	0.0427 (8)	
H112	0.1011	0.4202	0.5155	0.051*	
C113	0.20157 (13)	0.16831 (14)	0.19164 (13)	0.0267 (6)	
C114	0.26218 (14)	0.22995 (15)	0.23807 (14)	0.0306 (6)	
C115	0.19264 (9)	0.11448 (8)	0.21912 (10)	0.0300 (6)	
C116	0.24282 (8)	0.08072 (10)	0.22697 (10)	0.0407 (8)	
H116	0.2839	0.0935	0.2194	0.049*	
C117	0.23286 (10)	0.02820 (10)	0.24587 (11)	0.0488 (9)	
H117	0.2672	0.0051	0.2512	0.059*	
C118	0.17271 (11)	0.00945 (9)	0.25692 (11)	0.0476 (9)	
H118	0.1659	−0.0264	0.2698	0.057*	
C119	0.12252 (9)	0.04320 (10)	0.24907 (11)	0.0440 (8)	
H119	0.0814	0.0304	0.2566	0.053*	
C120	0.13249 (8)	0.09572 (10)	0.23018 (10)	0.0348 (7)	
H120	0.0982	0.1188	0.2248	0.042*	
C121	0.20064 (14)	0.13413 (15)	0.11772 (14)	0.0302 (6)	
C122	0.17908 (15)	0.06569 (17)	0.08276 (14)	0.0363 (7)	
H122	0.1647	0.0396	0.1046	0.044*	
C123	0.17812 (17)	0.03434 (18)	0.01602 (15)	0.0421 (8)	
H123	0.1630	−0.0127	−0.0072	0.051*	
C124	0.19894 (17)	0.07119 (19)	−0.01630 (15)	0.0446 (8)	
H124	0.1989	0.0497	−0.0616	0.054*	
C125	0.21982 (17)	0.13913 (18)	0.01714 (15)	0.0406 (8)	
H125	0.2340	0.1647	−0.0052	0.049*	
C126	0.22042 (15)	0.17098 (17)	0.08366 (14)	0.0344 (7)	
H126	0.2344	0.2182	0.1061	0.041*	
C127	0.32285 (15)	0.23608 (19)	0.21114 (15)	0.0379 (7)	
C128	0.35709 (18)	0.2994 (2)	0.22595 (19)	0.0531 (10)	
H128	0.3410	0.3374	0.2495	0.064*	
C129	0.4154 (2)	0.3080 (3)	0.2066 (2)	0.0728 (14)	
H129	0.4383	0.3517	0.2165	0.087*	
C130	0.4396 (2)	0.2531 (3)	0.1731 (2)	0.0730 (15)	
H130	0.4796	0.2590	0.1608	0.088*	
C131	0.40539 (19)	0.1898 (3)	0.15759 (18)	0.0617 (13)	
H131	0.4213	0.1518	0.1337	0.074*	
C132	0.34760 (17)	0.1815 (2)	0.17701 (16)	0.0472 (9)	
H132	0.3247	0.1377	0.1667	0.057*	
C133	0.28359 (14)	0.23236 (16)	0.30859 (14)	0.0325 (6)	
C134	0.23849 (15)	0.23868 (17)	0.34682 (15)	0.0357 (7)	
H134	0.1952	0.2425	0.3297	0.043*	
C135	0.25592 (16)	0.23949 (18)	0.40963 (15)	0.0416 (8)	
H135	0.2245	0.2438	0.4351	0.050*	
C136	0.31843 (17)	0.23402 (19)	0.43509 (16)	0.0438 (8)	
H136	0.3303	0.2344	0.4780	0.053*	
C137	0.36367 (17)	0.2280 (2)	0.39774 (17)	0.0505 (9)	
H137	0.4070	0.2244	0.4151	0.061*	
C138	0.34619 (16)	0.2270 (2)	0.33488 (16)	0.0448 (8)	
H138	0.3777	0.2226	0.3096	0.054*	
C139	0.11452 (14)	0.30685 (13)	0.11023 (13)	0.0249 (6)	
C140	0.14003 (15)	0.35849 (14)	0.09394 (14)	0.0302 (6)	
C141	0.09580 (16)	0.37419 (15)	0.04957 (14)	0.0333 (7)	
H141	0.1115	0.4082	0.0369	0.040*	
C142	0.03095 (16)	0.34220 (15)	0.02404 (13)	0.0313 (7)	
C143	0.00610 (15)	0.29394 (15)	0.04343 (13)	0.0286 (6)	
H143	−0.0393	0.2731	0.0272	0.034*	
C144	0.04787 (14)	0.27602 (13)	0.08684 (12)	0.0244 (6)	
C145	0.01722 (13)	0.22148 (13)	0.10247 (12)	0.0238 (5)	
C146	0.03493 (13)	0.15995 (14)	0.07974 (13)	0.0253 (6)	
H146	0.0682	0.1533	0.0564	0.030*	
C147	0.00396 (14)	0.10776 (14)	0.09116 (13)	0.0274 (6)	
C148	−0.04746 (14)	0.11583 (14)	0.12117 (13)	0.0283 (6)	
H148	−0.0693	0.0791	0.1268	0.034*	
C149	−0.06831 (14)	0.17682 (14)	0.14359 (13)	0.0271 (6)	
C150	−0.03293 (14)	0.23007 (13)	0.13598 (13)	0.0253 (6)	
C151	0.21271 (17)	0.39603 (16)	0.11998 (16)	0.0382 (7)	
C152	0.23336 (19)	0.42886 (19)	0.19566 (17)	0.0486 (9)	
H15A	0.2313	0.3942	0.2127	0.073*	
H15B	0.2035	0.4582	0.2119	0.073*	
H15C	0.2785	0.4552	0.2109	0.073*	
C153	0.2238 (2)	0.4533 (2)	0.0976 (2)	0.0572 (10)	
H15D	0.1946	0.4839	0.1126	0.086*	
H15E	0.2142	0.4347	0.0491	0.086*	
H15F	0.2698	0.4777	0.1168	0.086*	
C154	0.25774 (18)	0.34884 (19)	0.0937 (2)	0.0536 (10)	
H15G	0.3033	0.3741	0.1085	0.080*	
H15H	0.2440	0.3280	0.0452	0.080*	
H15I	0.2549	0.3141	0.1105	0.080*	
C155	0.0104 (2)	0.40039 (19)	−0.04448 (18)	0.0515 (10)	
H15J	0.0248	0.4444	−0.0071	0.077*	
H15K	−0.0250	0.4020	−0.0786	0.077*	
H15L	0.0476	0.3881	−0.0630	0.077*	
C156	−0.00040 (17)	−0.00332 (15)	0.08294 (17)	0.0402 (7)	
H15M	0.0030	0.0120	0.1304	0.060*	
H15N	0.0231	−0.0398	0.0689	0.060*	
H15O	−0.0469	−0.0193	0.0582	0.060*	
C157	−0.12895 (15)	0.18259 (15)	0.17211 (15)	0.0333 (6)	
C158	−0.16003 (18)	0.11615 (17)	0.17183 (18)	0.0461 (8)	
H15P	−0.1731	0.0815	0.1264	0.069*	
H15Q	−0.1989	0.1215	0.1893	0.069*	
H15R	−0.1279	0.1030	0.1996	0.069*	
C159	−0.18161 (16)	0.2013 (2)	0.12850 (18)	0.0481 (9)	
H15S	−0.1915	0.1686	0.0824	0.072*	
H15T	−0.1650	0.2459	0.1314	0.072*	
H15U	−0.2218	0.2014	0.1440	0.072*	
C160	−0.11179 (17)	0.23403 (16)	0.24380 (15)	0.0387 (7)	
H16A	−0.1523	0.2395	0.2584	0.058*	
H16B	−0.0893	0.2770	0.2471	0.058*	
H16C	−0.0827	0.2187	0.2720	0.058*	
C161	−0.03418 (15)	0.45161 (14)	0.17801 (14)	0.0296 (6)	
C162	−0.06867 (16)	0.45134 (15)	0.23706 (14)	0.0339 (7)	
C163	0.00807 (16)	0.51957 (15)	0.19385 (14)	0.0326 (7)	
C164	−0.01801 (17)	0.57729 (15)	0.21039 (14)	0.0351 (7)	
H164	−0.0627	0.5745	0.2123	0.042*	
C165	0.0205 (2)	0.63833 (16)	0.22394 (16)	0.0451 (8)	
H165	0.0020	0.6770	0.2350	0.054*	
C166	0.0854 (2)	0.64360 (18)	0.22156 (19)	0.0510 (9)	
H166	0.1121	0.6857	0.2319	0.061*	
C167	0.1110 (2)	0.58625 (18)	0.20382 (19)	0.0513 (9)	
H167	0.1554	0.5891	0.2010	0.062*	
C168	0.07275 (17)	0.52500 (16)	0.19012 (16)	0.0384 (7)	
H168	0.0912	0.4863	0.1780	0.046*	
C169	−0.08256 (15)	0.42383 (15)	0.10878 (14)	0.0315 (6)	
C170	−0.12519 (16)	0.36185 (17)	0.08348 (17)	0.0404 (7)	
H170	−0.1243	0.3364	0.1092	0.048*	
C171	−0.16906 (18)	0.33647 (19)	0.02126 (19)	0.0496 (9)	
H171	−0.1972	0.2937	0.0047	0.059*	
C172	−0.1721 (2)	0.3727 (2)	−0.01670 (18)	0.0519 (9)	
H172	−0.2029	0.3558	−0.0588	0.062*	
C173	−0.12977 (19)	0.43401 (19)	0.00750 (16)	0.0459 (9)	
H173	−0.1312	0.4593	−0.0184	0.055*	
C174	−0.08501 (17)	0.45926 (16)	0.06938 (14)	0.0369 (7)	
H174	−0.0557	0.5012	0.0849	0.044*	
C175	−0.04194 (11)	0.51649 (8)	0.30102 (9)	0.0416 (8)	
C176	0.02482 (11)	0.53268 (10)	0.33272 (10)	0.0450 (9)	
H176	0.0527	0.5036	0.3155	0.054*	
C177	0.05072 (12)	0.59142 (11)	0.38958 (10)	0.0607 (11)	
H177	0.0963	0.6025	0.4112	0.073*	
C178	0.00987 (16)	0.63398 (9)	0.41475 (9)	0.0757 (16)	
H178	0.0276	0.6741	0.4536	0.091*	
C179	−0.05689 (15)	0.61779 (11)	0.38305 (12)	0.0752 (16)	
H179	−0.0848	0.6469	0.4003	0.090*	
C180	−0.08280 (11)	0.55904 (12)	0.32619 (11)	0.0553 (11)	
H180	−0.1284	0.5480	0.3045	0.066*	
O1	0.52189 (12)	0.80423 (14)	0.24419 (13)	0.0511 (6)	
O2	0.70751 (9)	0.75800 (10)	0.22791 (9)	0.0278 (4)	
O3	0.79497 (9)	0.85067 (9)	0.26313 (9)	0.0242 (4)	
O4	0.70120 (10)	0.82558 (10)	0.16318 (9)	0.0279 (4)	
O5	0.60947 (13)	0.96466 (13)	0.01826 (11)	0.0490 (6)	
O6	0.93927 (11)	0.98817 (11)	0.26420 (13)	0.0479 (6)	
O7	0.59318 (9)	1.03691 (10)	0.31967 (9)	0.0291 (4)	
O8	0.57541 (9)	0.97188 (10)	0.20592 (9)	0.0284 (4)	
O9	0.69029 (9)	1.03652 (10)	0.27378 (9)	0.0272 (4)	
O10	0.18001 (15)	0.48054 (13)	0.33593 (13)	0.0581 (7)	
O11	0.14430 (9)	0.19901 (9)	0.19634 (9)	0.0245 (4)	
O12	0.23554 (9)	0.28976 (10)	0.24581 (10)	0.0296 (4)	
O13	0.15871 (9)	0.28411 (10)	0.14680 (9)	0.0266 (4)	
O14	−0.01313 (12)	0.35224 (11)	−0.02241 (10)	0.0406 (5)	
O15	0.02806 (11)	0.05070 (10)	0.07027 (11)	0.0349 (5)	
O16	0.01124 (10)	0.40691 (10)	0.17689 (9)	0.0291 (4)	
O17	−0.04602 (10)	0.39883 (10)	0.25383 (9)	0.0299 (4)	
O18	−0.04884 (9)	0.29258 (9)	0.16054 (9)	0.0270 (4)	
P1	0.66318 (4)	0.91475 (4)	0.40864 (3)	0.02728 (16)	
P2	0.71557 (3)	0.83615 (4)	0.24085 (3)	0.02297 (14)	
P3	0.62843 (3)	0.98398 (4)	0.27309 (3)	0.02525 (15)	
P4	0.08712 (4)	0.33859 (4)	0.37468 (3)	0.02542 (15)	
P5	0.00459 (4)	0.35991 (4)	0.21620 (3)	0.02480 (15)	
P6	0.15651 (3)	0.27847 (4)	0.21677 (3)	0.02324 (14)	
Rh1	0.656131 (10)	0.900667 (11)	0.300209 (10)	0.02435 (6)	
Rh2	0.093304 (11)	0.340364 (10)	0.273920 (10)	0.02364 (6)	
O20	0.1879 (3)	0.7752 (3)	0.3581 (3)	0.0785 (18)*	0.50
C192	0.1299 (5)	0.8518 (4)	0.3392 (5)	0.072 (2)*	0.50
H19D	0.0942	0.8754	0.3484	0.108*	0.50
H19E	0.1209	0.8249	0.2915	0.108*	0.50
H19F	0.1716	0.8843	0.3544	0.108*	0.50
C191	0.1348 (5)	0.8064 (5)	0.3754 (5)	0.090 (3)*	0.50
H19G	0.1445	0.8326	0.4238	0.108*	0.50
H19H	0.0933	0.7727	0.3605	0.108*	0.50
C193	0.1918 (5)	0.7289 (5)	0.3884 (5)	0.080 (3)*	0.50
H19I	0.1503	0.6949	0.3709	0.096*	0.50
H19J	0.1996	0.7526	0.4370	0.096*	0.50
C194	0.2489 (7)	0.6960 (8)	0.3717 (8)	0.149 (6)*	0.50
H19K	0.2531	0.6635	0.3912	0.223*	0.50
H19L	0.2898	0.7302	0.3897	0.223*	0.50
H19M	0.2408	0.6732	0.3235	0.223*	0.50
H1	0.7142 (18)	0.9498 (19)	0.3316 (18)	0.059 (11)*	
H2	0.0502 (18)	0.2781 (18)	0.2480 (18)	0.053 (10)*	

### Atomic displacement parameters (Å^2^)

**Table:**

	*U*^11^	*U*^22^	*U*^33^	*U*^12^	*U*^13^	*U*^23^
C181	0.0609 (15)	0.121 (2)	0.118 (2)	0.0685 (15)	0.0693 (15)	0.1035 (18)
C182	0.0620 (15)	0.122 (2)	0.117 (2)	0.0701 (15)	0.0668 (15)	0.1016 (18)
C183	0.0664 (16)	0.135 (2)	0.136 (2)	0.0651 (16)	0.0603 (16)	0.1062 (19)
C184	0.0700 (17)	0.146 (2)	0.156 (2)	0.0570 (17)	0.0570 (18)	0.108 (2)
C185	0.0713 (17)	0.145 (2)	0.156 (2)	0.0539 (17)	0.0593 (18)	0.112 (2)
C186	0.0657 (16)	0.134 (2)	0.141 (2)	0.0604 (16)	0.0646 (16)	0.1103 (19)
O19	0.076 (4)	0.057 (3)	0.050 (3)	0.003 (3)	0.008 (3)	0.023 (3)
C187	0.046 (4)	0.087 (5)	0.049 (4)	0.000 (4)	0.009 (3)	−0.019 (4)
C188	0.045 (4)	0.078 (5)	0.054 (4)	0.013 (3)	0.000 (3)	−0.001 (4)
C189	0.109 (7)	0.083 (6)	0.108 (7)	0.008 (5)	0.051 (6)	0.039 (5)
C190	0.119 (7)	0.086 (6)	0.105 (7)	0.014 (6)	0.038 (6)	0.041 (5)
C1	0.0407 (18)	0.0461 (18)	0.0263 (15)	0.0199 (15)	0.0142 (13)	0.0196 (14)
C2	0.0409 (16)	0.0474 (17)	0.0225 (14)	0.0272 (14)	0.0195 (12)	0.0220 (13)
C3	0.0405 (17)	0.057 (2)	0.0284 (15)	0.0284 (16)	0.0149 (13)	0.0217 (15)
C4	0.049 (2)	0.071 (2)	0.0385 (18)	0.0440 (19)	0.0232 (16)	0.0289 (18)
C5	0.070 (2)	0.057 (2)	0.0386 (19)	0.043 (2)	0.0303 (18)	0.0261 (17)
C6	0.058 (2)	0.052 (2)	0.0368 (18)	0.0292 (18)	0.0206 (16)	0.0187 (16)
C7	0.0407 (17)	0.0500 (19)	0.0315 (16)	0.0234 (15)	0.0176 (14)	0.0213 (15)
C8	0.0360 (16)	0.0509 (18)	0.0315 (16)	0.0242 (14)	0.0175 (13)	0.0289 (14)
C9	0.0427 (18)	0.061 (2)	0.0394 (18)	0.0313 (16)	0.0236 (14)	0.0360 (16)
C10	0.0444 (19)	0.086 (3)	0.056 (2)	0.0340 (19)	0.0300 (17)	0.054 (2)
C11	0.052 (2)	0.076 (3)	0.067 (3)	0.018 (2)	0.0246 (19)	0.054 (2)
C12	0.065 (2)	0.056 (2)	0.055 (2)	0.0167 (19)	0.0232 (19)	0.0324 (19)
C13	0.0453 (19)	0.056 (2)	0.0376 (18)	0.0211 (16)	0.0200 (15)	0.0294 (16)
C14	0.0359 (16)	0.0439 (17)	0.0233 (14)	0.0264 (14)	0.0101 (12)	0.0147 (13)
C15	0.055 (2)	0.052 (2)	0.0304 (16)	0.0295 (17)	0.0125 (15)	0.0226 (15)
C16	0.075 (3)	0.058 (2)	0.0282 (17)	0.037 (2)	0.0015 (17)	0.0183 (16)
C17	0.050 (2)	0.060 (2)	0.045 (2)	0.0329 (19)	−0.0060 (17)	0.0110 (18)
C18	0.0366 (18)	0.063 (2)	0.048 (2)	0.0237 (17)	0.0061 (16)	0.0166 (18)
C19	0.0351 (17)	0.057 (2)	0.0337 (17)	0.0265 (15)	0.0114 (13)	0.0177 (15)
C20	0.0355 (15)	0.0343 (15)	0.0280 (14)	0.0214 (13)	0.0175 (12)	0.0199 (12)
C21	0.0324 (14)	0.0299 (14)	0.0267 (14)	0.0206 (12)	0.0153 (11)	0.0178 (12)
C22	0.0368 (16)	0.0377 (16)	0.0277 (15)	0.0193 (13)	0.0166 (12)	0.0212 (13)
C23	0.0391 (16)	0.0371 (16)	0.0294 (15)	0.0187 (13)	0.0157 (13)	0.0198 (13)
C24	0.0489 (19)	0.0472 (18)	0.0298 (16)	0.0233 (15)	0.0211 (14)	0.0223 (14)
C25	0.060 (2)	0.052 (2)	0.0330 (17)	0.0222 (17)	0.0205 (15)	0.0306 (16)
C26	0.081 (3)	0.0427 (19)	0.0395 (19)	0.0265 (18)	0.0275 (18)	0.0296 (16)
C27	0.069 (2)	0.0379 (17)	0.0326 (17)	0.0238 (16)	0.0250 (16)	0.0206 (14)
C28	0.0499 (18)	0.0335 (15)	0.0267 (15)	0.0192 (14)	0.0207 (13)	0.0202 (13)
C29	0.054 (2)	0.0406 (18)	0.0350 (17)	0.0171 (16)	0.0156 (15)	0.0176 (14)
C30	0.076 (3)	0.044 (2)	0.0353 (19)	0.0112 (19)	0.0073 (18)	0.0142 (16)
C31	0.096 (3)	0.0365 (18)	0.0358 (19)	0.026 (2)	0.034 (2)	0.0168 (15)
C32	0.075 (2)	0.0365 (17)	0.0366 (18)	0.0293 (17)	0.0317 (18)	0.0210 (15)
C33	0.055 (2)	0.0360 (16)	0.0351 (17)	0.0249 (15)	0.0243 (15)	0.0209 (14)
C34	0.0375 (16)	0.0328 (15)	0.0308 (15)	0.0199 (13)	0.0205 (12)	0.0191 (12)
C35	0.0427 (17)	0.0370 (16)	0.0303 (15)	0.0204 (14)	0.0206 (13)	0.0181 (13)
C36	0.059 (2)	0.0450 (18)	0.0279 (16)	0.0231 (16)	0.0249 (15)	0.0171 (14)
C37	0.064 (2)	0.0435 (18)	0.046 (2)	0.0289 (17)	0.0417 (18)	0.0217 (16)
C38	0.050 (2)	0.053 (2)	0.054 (2)	0.0325 (17)	0.0356 (17)	0.0309 (17)
C39	0.0446 (18)	0.055 (2)	0.0442 (19)	0.0326 (16)	0.0262 (15)	0.0332 (16)
C40	0.0295 (14)	0.0429 (16)	0.0263 (14)	0.0191 (13)	0.0155 (11)	0.0223 (13)
C41	0.0361 (16)	0.0475 (18)	0.0370 (17)	0.0214 (14)	0.0165 (13)	0.0273 (15)
C42	0.0410 (18)	0.076 (3)	0.053 (2)	0.0300 (18)	0.0165 (16)	0.049 (2)
C43	0.0369 (18)	0.078 (3)	0.043 (2)	0.0192 (18)	0.0040 (15)	0.034 (2)
C44	0.0378 (18)	0.053 (2)	0.0430 (19)	0.0144 (16)	0.0064 (15)	0.0189 (16)
C45	0.0343 (16)	0.0454 (18)	0.0355 (16)	0.0208 (14)	0.0127 (13)	0.0219 (14)
C46	0.0323 (14)	0.0442 (17)	0.0199 (13)	0.0216 (13)	0.0145 (11)	0.0210 (12)
C47	0.0339 (15)	0.0491 (18)	0.0212 (14)	0.0199 (14)	0.0137 (12)	0.0178 (13)
C48	0.0398 (17)	0.0525 (19)	0.0204 (14)	0.0209 (15)	0.0088 (12)	0.0188 (14)
C49	0.0460 (18)	0.059 (2)	0.0245 (15)	0.0318 (16)	0.0160 (13)	0.0275 (15)
C50	0.0414 (16)	0.0386 (16)	0.0252 (14)	0.0226 (13)	0.0172 (12)	0.0190 (13)
C51	0.0318 (14)	0.0413 (16)	0.0194 (13)	0.0221 (13)	0.0154 (11)	0.0179 (12)
C52	0.0327 (14)	0.0342 (15)	0.0236 (13)	0.0160 (12)	0.0142 (11)	0.0205 (12)
C53	0.0358 (15)	0.0301 (14)	0.0315 (15)	0.0166 (12)	0.0196 (12)	0.0194 (12)
C54	0.0266 (14)	0.0334 (15)	0.0483 (18)	0.0160 (12)	0.0176 (13)	0.0233 (14)
C55	0.0293 (15)	0.0330 (15)	0.0400 (17)	0.0110 (12)	0.0108 (13)	0.0175 (13)
C56	0.0342 (15)	0.0327 (15)	0.0284 (14)	0.0153 (12)	0.0137 (12)	0.0198 (12)
C57	0.0277 (14)	0.0357 (15)	0.0287 (14)	0.0179 (12)	0.0158 (11)	0.0226 (12)
C58	0.0424 (18)	0.0500 (19)	0.0231 (15)	0.0134 (15)	0.0080 (13)	0.0151 (14)
C59	0.053 (2)	0.067 (3)	0.037 (2)	0.0063 (19)	0.0002 (17)	0.0216 (18)
C60	0.059 (2)	0.049 (2)	0.0350 (18)	0.0186 (17)	0.0185 (16)	0.0151 (16)
C61	0.046 (2)	0.057 (2)	0.0349 (18)	0.0040 (17)	0.0125 (15)	0.0210 (16)
C62	0.075 (3)	0.076 (3)	0.055 (2)	0.042 (2)	0.029 (2)	0.051 (2)
C63	0.0404 (19)	0.048 (2)	0.085 (3)	0.0241 (17)	0.040 (2)	0.025 (2)
C64	0.0345 (15)	0.0347 (16)	0.0290 (15)	0.0136 (13)	0.0116 (12)	0.0151 (13)
C65	0.046 (2)	0.047 (2)	0.0424 (19)	0.0148 (16)	0.0108 (16)	0.0112 (16)
C66	0.059 (2)	0.0374 (18)	0.0428 (19)	0.0224 (16)	0.0139 (16)	0.0180 (15)
C67	0.056 (2)	0.048 (2)	0.0325 (17)	0.0127 (17)	0.0229 (16)	0.0129 (15)
C68	0.0300 (14)	0.0508 (18)	0.0247 (14)	0.0284 (14)	0.0137 (11)	0.0221 (13)
C69	0.0305 (14)	0.0445 (17)	0.0246 (14)	0.0247 (13)	0.0144 (11)	0.0209 (13)
C70	0.0365 (16)	0.0479 (18)	0.0295 (15)	0.0257 (14)	0.0155 (13)	0.0185 (14)
C71	0.049 (2)	0.057 (2)	0.0358 (18)	0.0295 (17)	0.0150 (15)	0.0197 (16)
C72	0.062 (2)	0.056 (2)	0.045 (2)	0.022 (2)	0.0105 (18)	0.0102 (18)
C73	0.064 (3)	0.044 (2)	0.067 (3)	0.0252 (19)	0.020 (2)	0.0178 (19)
C74	0.050 (2)	0.053 (2)	0.056 (2)	0.0299 (18)	0.0175 (17)	0.0269 (18)
C75	0.0416 (18)	0.052 (2)	0.0358 (17)	0.0290 (16)	0.0154 (14)	0.0209 (15)
C76	0.0282 (15)	0.071 (2)	0.0223 (14)	0.0231 (15)	0.0111 (12)	0.0253 (15)
C77	0.0364 (17)	0.085 (3)	0.0270 (16)	0.0288 (18)	0.0145 (14)	0.0237 (17)
C78	0.0369 (19)	0.121 (4)	0.0335 (19)	0.027 (2)	0.0194 (15)	0.033 (2)
C79	0.038 (2)	0.146 (5)	0.055 (3)	0.016 (3)	0.0172 (18)	0.066 (3)
C80	0.041 (2)	0.102 (3)	0.067 (3)	0.012 (2)	0.0089 (19)	0.062 (3)
C81	0.0364 (17)	0.069 (2)	0.0442 (19)	0.0195 (17)	0.0119 (15)	0.0368 (18)
C82	0.0351 (16)	0.0506 (18)	0.0295 (15)	0.0307 (14)	0.0197 (13)	0.0247 (14)
C83	0.0425 (18)	0.0520 (19)	0.0403 (18)	0.0294 (16)	0.0205 (14)	0.0306 (16)
C84	0.050 (2)	0.059 (2)	0.062 (2)	0.0271 (18)	0.0256 (18)	0.041 (2)
C85	0.071 (3)	0.083 (3)	0.072 (3)	0.045 (2)	0.042 (2)	0.062 (3)
C86	0.057 (2)	0.094 (3)	0.046 (2)	0.044 (2)	0.0275 (18)	0.052 (2)
C87	0.0414 (17)	0.068 (2)	0.0326 (16)	0.0323 (17)	0.0208 (14)	0.0314 (16)
C88	0.0323 (15)	0.0545 (19)	0.0252 (15)	0.0219 (14)	0.0118 (12)	0.0223 (14)
C89	0.0379 (17)	0.062 (2)	0.0246 (15)	0.0278 (16)	0.0144 (13)	0.0213 (15)
C90	0.0356 (18)	0.088 (3)	0.0340 (18)	0.0278 (18)	0.0147 (14)	0.0298 (19)
C91	0.036 (2)	0.078 (3)	0.058 (3)	0.009 (2)	0.0026 (18)	0.021 (2)
C92	0.052 (2)	0.064 (3)	0.069 (3)	0.016 (2)	0.001 (2)	0.015 (2)
C93	0.0393 (19)	0.058 (2)	0.049 (2)	0.0237 (17)	0.0073 (16)	0.0171 (18)
C94	0.0490 (19)	0.0381 (18)	0.0285 (16)	0.0131 (15)	0.0219 (14)	0.0154 (14)
C95	0.0362 (15)	0.0338 (15)	0.0322 (15)	0.0215 (13)	0.0237 (13)	0.0212 (13)
C96	0.0428 (17)	0.0465 (18)	0.0378 (17)	0.0253 (15)	0.0214 (14)	0.0287 (15)
C97	0.0469 (19)	0.060 (2)	0.055 (2)	0.0274 (17)	0.0243 (17)	0.0467 (19)
C98	0.0460 (19)	0.047 (2)	0.073 (3)	0.0251 (16)	0.0301 (18)	0.045 (2)
C99	0.0453 (19)	0.0349 (17)	0.057 (2)	0.0178 (15)	0.0260 (16)	0.0237 (16)
C100	0.0459 (18)	0.0324 (16)	0.0352 (16)	0.0173 (14)	0.0216 (14)	0.0177 (13)
C101	0.0463 (17)	0.0374 (16)	0.0197 (13)	0.0239 (14)	0.0200 (12)	0.0169 (12)
C102	0.0447 (17)	0.0325 (15)	0.0368 (16)	0.0200 (14)	0.0231 (14)	0.0185 (13)
C103	0.0460 (19)	0.0469 (19)	0.050 (2)	0.0223 (16)	0.0297 (16)	0.0243 (16)
C104	0.052 (2)	0.053 (2)	0.0449 (19)	0.0348 (17)	0.0319 (16)	0.0270 (16)
C105	0.069 (2)	0.0444 (19)	0.0454 (19)	0.0391 (18)	0.0366 (18)	0.0294 (16)
C106	0.0551 (19)	0.0400 (17)	0.0359 (16)	0.0274 (15)	0.0309 (15)	0.0248 (14)
C107	0.0533 (19)	0.0327 (15)	0.0231 (14)	0.0180 (14)	0.0133 (13)	0.0151 (12)
C108	0.056 (2)	0.053 (2)	0.0327 (18)	0.0071 (17)	0.0129 (16)	0.0198 (16)
C109	0.061 (3)	0.076 (3)	0.056 (3)	−0.006 (2)	0.002 (2)	0.035 (2)
C110	0.098 (4)	0.046 (2)	0.037 (2)	−0.004 (2)	−0.011 (2)	0.0163 (18)
C111	0.098 (3)	0.0384 (19)	0.0249 (17)	0.029 (2)	0.0071 (19)	0.0102 (14)
C112	0.070 (2)	0.0406 (18)	0.0272 (16)	0.0324 (17)	0.0151 (15)	0.0175 (14)
C113	0.0287 (14)	0.0385 (15)	0.0242 (14)	0.0237 (12)	0.0140 (11)	0.0169 (12)
C114	0.0322 (15)	0.0459 (17)	0.0269 (15)	0.0230 (13)	0.0166 (12)	0.0206 (13)
C115	0.0421 (16)	0.0384 (16)	0.0169 (13)	0.0239 (13)	0.0114 (12)	0.0131 (12)
C116	0.0499 (19)	0.0492 (19)	0.0361 (17)	0.0312 (16)	0.0166 (15)	0.0230 (15)
C117	0.072 (2)	0.053 (2)	0.0348 (18)	0.0419 (19)	0.0136 (17)	0.0245 (16)
C118	0.081 (3)	0.0444 (19)	0.0333 (18)	0.0318 (19)	0.0202 (17)	0.0245 (15)
C119	0.064 (2)	0.0445 (19)	0.0392 (18)	0.0245 (17)	0.0234 (16)	0.0249 (16)
C120	0.0453 (18)	0.0415 (17)	0.0306 (16)	0.0237 (14)	0.0170 (13)	0.0209 (14)
C121	0.0329 (15)	0.0447 (17)	0.0255 (14)	0.0259 (13)	0.0157 (12)	0.0189 (13)
C122	0.0410 (17)	0.0510 (19)	0.0270 (15)	0.0246 (15)	0.0180 (13)	0.0180 (14)
C123	0.0490 (19)	0.049 (2)	0.0268 (16)	0.0207 (16)	0.0156 (14)	0.0088 (14)
C124	0.050 (2)	0.066 (2)	0.0228 (15)	0.0302 (18)	0.0187 (14)	0.0144 (15)
C125	0.0503 (19)	0.061 (2)	0.0316 (16)	0.0344 (17)	0.0244 (15)	0.0290 (16)
C126	0.0387 (16)	0.0501 (18)	0.0273 (15)	0.0284 (15)	0.0164 (13)	0.0206 (14)
C127	0.0298 (15)	0.071 (2)	0.0287 (16)	0.0232 (16)	0.0152 (12)	0.0298 (16)
C128	0.042 (2)	0.084 (3)	0.059 (2)	0.024 (2)	0.0257 (18)	0.048 (2)
C129	0.045 (2)	0.122 (4)	0.089 (3)	0.019 (2)	0.029 (2)	0.078 (3)
C130	0.043 (2)	0.159 (5)	0.067 (3)	0.048 (3)	0.038 (2)	0.080 (3)
C131	0.044 (2)	0.131 (4)	0.040 (2)	0.052 (3)	0.0259 (17)	0.050 (2)
C132	0.0417 (18)	0.090 (3)	0.0295 (17)	0.0379 (19)	0.0197 (14)	0.0329 (18)
C133	0.0316 (15)	0.0476 (18)	0.0267 (15)	0.0174 (14)	0.0126 (12)	0.0192 (13)
C134	0.0328 (16)	0.0545 (19)	0.0279 (15)	0.0203 (14)	0.0132 (13)	0.0198 (14)
C135	0.0412 (18)	0.070 (2)	0.0273 (16)	0.0264 (17)	0.0203 (14)	0.0251 (16)
C136	0.0445 (19)	0.073 (2)	0.0258 (16)	0.0245 (17)	0.0130 (14)	0.0272 (16)
C137	0.0407 (19)	0.092 (3)	0.0358 (18)	0.0309 (19)	0.0137 (15)	0.0376 (19)
C138	0.0359 (17)	0.082 (3)	0.0324 (17)	0.0258 (17)	0.0179 (14)	0.0327 (18)
C139	0.0374 (15)	0.0291 (14)	0.0199 (13)	0.0179 (12)	0.0173 (11)	0.0145 (11)
C140	0.0437 (17)	0.0331 (15)	0.0264 (14)	0.0175 (13)	0.0225 (13)	0.0167 (12)
C141	0.056 (2)	0.0320 (15)	0.0288 (15)	0.0221 (14)	0.0275 (14)	0.0192 (13)
C142	0.0500 (18)	0.0410 (16)	0.0210 (14)	0.0282 (15)	0.0225 (13)	0.0205 (12)
C143	0.0365 (15)	0.0400 (16)	0.0228 (14)	0.0229 (13)	0.0187 (12)	0.0178 (12)
C144	0.0388 (15)	0.0277 (13)	0.0191 (13)	0.0188 (12)	0.0205 (11)	0.0134 (11)
C145	0.0305 (14)	0.0314 (14)	0.0160 (12)	0.0132 (11)	0.0096 (10)	0.0129 (11)
C146	0.0298 (14)	0.0339 (15)	0.0198 (13)	0.0148 (12)	0.0122 (11)	0.0143 (11)
C147	0.0319 (15)	0.0307 (14)	0.0243 (14)	0.0132 (12)	0.0095 (11)	0.0134 (12)
C148	0.0349 (15)	0.0299 (14)	0.0251 (14)	0.0081 (12)	0.0113 (12)	0.0145 (12)
C149	0.0285 (14)	0.0376 (15)	0.0194 (13)	0.0103 (12)	0.0110 (11)	0.0131 (12)
C150	0.0347 (15)	0.0295 (14)	0.0195 (13)	0.0164 (12)	0.0131 (11)	0.0128 (11)
C151	0.0514 (19)	0.0390 (17)	0.0353 (17)	0.0104 (15)	0.0225 (15)	0.0216 (14)
C152	0.057 (2)	0.050 (2)	0.0402 (19)	0.0036 (17)	0.0167 (17)	0.0205 (17)
C153	0.066 (3)	0.064 (2)	0.058 (2)	0.005 (2)	0.025 (2)	0.041 (2)
C154	0.044 (2)	0.056 (2)	0.061 (2)	0.0078 (17)	0.0313 (18)	0.0175 (19)
C155	0.080 (3)	0.068 (2)	0.045 (2)	0.043 (2)	0.0354 (19)	0.0468 (19)
C156	0.053 (2)	0.0310 (16)	0.0438 (19)	0.0143 (15)	0.0149 (15)	0.0199 (14)
C157	0.0358 (16)	0.0400 (17)	0.0297 (15)	0.0115 (13)	0.0189 (13)	0.0145 (13)
C158	0.051 (2)	0.047 (2)	0.047 (2)	0.0067 (16)	0.0305 (17)	0.0178 (16)
C159	0.0359 (18)	0.075 (3)	0.048 (2)	0.0199 (17)	0.0197 (15)	0.0332 (19)
C160	0.0478 (19)	0.0448 (18)	0.0331 (17)	0.0148 (15)	0.0254 (14)	0.0177 (14)
C161	0.0469 (17)	0.0334 (15)	0.0285 (14)	0.0293 (13)	0.0255 (13)	0.0207 (12)
C162	0.0541 (19)	0.0406 (16)	0.0315 (15)	0.0337 (15)	0.0296 (14)	0.0253 (14)
C163	0.0529 (19)	0.0356 (16)	0.0242 (14)	0.0226 (14)	0.0206 (13)	0.0193 (13)
C164	0.0551 (19)	0.0395 (17)	0.0265 (15)	0.0267 (15)	0.0196 (14)	0.0211 (13)
C165	0.077 (3)	0.0346 (17)	0.0343 (17)	0.0279 (17)	0.0217 (17)	0.0170 (14)
C166	0.071 (3)	0.0356 (18)	0.050 (2)	0.0115 (18)	0.0214 (19)	0.0195 (16)
C167	0.056 (2)	0.046 (2)	0.060 (2)	0.0163 (18)	0.0272 (19)	0.0241 (18)
C168	0.0508 (19)	0.0379 (17)	0.0375 (17)	0.0202 (15)	0.0221 (15)	0.0190 (14)
C169	0.0433 (17)	0.0396 (16)	0.0300 (15)	0.0274 (14)	0.0235 (13)	0.0217 (13)
C170	0.0426 (18)	0.0448 (19)	0.0460 (19)	0.0203 (15)	0.0141 (15)	0.0272 (16)
C171	0.047 (2)	0.051 (2)	0.053 (2)	0.0175 (17)	0.0094 (17)	0.0229 (18)
C172	0.060 (2)	0.066 (3)	0.0357 (19)	0.030 (2)	0.0115 (17)	0.0229 (18)
C173	0.067 (2)	0.059 (2)	0.0293 (17)	0.0350 (19)	0.0222 (16)	0.0260 (16)
C174	0.059 (2)	0.0413 (17)	0.0263 (15)	0.0279 (16)	0.0236 (14)	0.0195 (13)
C175	0.084 (3)	0.0366 (17)	0.0342 (17)	0.0375 (17)	0.0426 (18)	0.0264 (14)
C176	0.083 (3)	0.0365 (17)	0.0323 (17)	0.0285 (18)	0.0303 (18)	0.0206 (14)
C177	0.115 (4)	0.045 (2)	0.0317 (19)	0.024 (2)	0.024 (2)	0.0209 (16)
C178	0.174 (5)	0.041 (2)	0.032 (2)	0.050 (3)	0.044 (3)	0.0204 (17)
C179	0.164 (5)	0.061 (3)	0.046 (2)	0.075 (3)	0.069 (3)	0.036 (2)
C180	0.108 (3)	0.053 (2)	0.043 (2)	0.054 (2)	0.053 (2)	0.0335 (18)
O1	0.0388 (14)	0.0633 (17)	0.0517 (15)	0.0057 (13)	0.0115 (12)	0.0259 (13)
O2	0.0296 (10)	0.0352 (11)	0.0291 (10)	0.0170 (9)	0.0132 (8)	0.0187 (9)
O3	0.0290 (10)	0.0297 (10)	0.0243 (9)	0.0179 (8)	0.0131 (8)	0.0159 (8)
O4	0.0359 (11)	0.0397 (11)	0.0201 (9)	0.0217 (9)	0.0134 (8)	0.0184 (8)
O5	0.0640 (16)	0.0652 (16)	0.0346 (13)	0.0304 (13)	0.0087 (11)	0.0356 (12)
O6	0.0279 (11)	0.0389 (13)	0.0750 (17)	0.0166 (10)	0.0203 (11)	0.0154 (12)
O7	0.0301 (10)	0.0478 (12)	0.0201 (9)	0.0259 (9)	0.0109 (8)	0.0181 (9)
O8	0.0289 (10)	0.0435 (11)	0.0239 (10)	0.0235 (9)	0.0129 (8)	0.0182 (9)
O9	0.0282 (10)	0.0383 (11)	0.0272 (10)	0.0202 (9)	0.0132 (8)	0.0199 (9)
O10	0.082 (2)	0.0387 (14)	0.0507 (16)	0.0007 (14)	0.0295 (14)	0.0134 (12)
O11	0.0275 (10)	0.0329 (10)	0.0239 (9)	0.0185 (8)	0.0138 (8)	0.0165 (8)
O12	0.0287 (10)	0.0367 (11)	0.0303 (11)	0.0149 (9)	0.0120 (8)	0.0167 (9)
O13	0.0346 (10)	0.0356 (11)	0.0235 (10)	0.0194 (9)	0.0169 (8)	0.0189 (8)
O14	0.0606 (14)	0.0544 (14)	0.0345 (12)	0.0354 (12)	0.0264 (11)	0.0343 (11)
O15	0.0443 (12)	0.0293 (11)	0.0412 (12)	0.0169 (9)	0.0201 (10)	0.0184 (9)
O16	0.0439 (12)	0.0342 (11)	0.0295 (10)	0.0266 (9)	0.0247 (9)	0.0220 (9)
O17	0.0474 (12)	0.0356 (11)	0.0282 (10)	0.0283 (9)	0.0261 (9)	0.0225 (9)
O18	0.0344 (10)	0.0332 (10)	0.0255 (10)	0.0193 (9)	0.0168 (8)	0.0170 (8)
P1	0.0307 (4)	0.0428 (4)	0.0218 (3)	0.0228 (3)	0.0146 (3)	0.0197 (3)
P2	0.0281 (4)	0.0322 (4)	0.0192 (3)	0.0166 (3)	0.0120 (3)	0.0160 (3)
P3	0.0261 (3)	0.0400 (4)	0.0210 (3)	0.0202 (3)	0.0116 (3)	0.0182 (3)
P4	0.0376 (4)	0.0287 (4)	0.0208 (3)	0.0179 (3)	0.0170 (3)	0.0140 (3)
P5	0.0377 (4)	0.0285 (4)	0.0216 (3)	0.0200 (3)	0.0188 (3)	0.0156 (3)
P6	0.0282 (3)	0.0308 (4)	0.0195 (3)	0.0147 (3)	0.0129 (3)	0.0142 (3)
Rh1	0.02663 (11)	0.03804 (13)	0.02029 (11)	0.01886 (9)	0.01278 (8)	0.01802 (9)
Rh2	0.03381 (12)	0.02724 (11)	0.01958 (11)	0.01581 (9)	0.01563 (9)	0.01313 (9)

### Geometric parameters (Å, º)

**Table:**

C181—C182	1.3900	C92—H92	0.9500
C181—C186	1.3900	C93—H93	0.9500
C181—C162	1.528 (4)	C94—O10	1.145 (4)
C182—C183	1.3900	C94—Rh2	1.907 (3)
C182—H182	0.9500	C95—C96	1.3900
C183—C184	1.3900	C95—C100	1.3900
C183—H183	0.9500	C95—P4	1.8455 (14)
C184—C185	1.3900	C96—C97	1.3900
C184—H184	0.9500	C96—H96	0.9500
C185—C186	1.3900	C97—C98	1.3900
C185—H185	0.9500	C97—H97	0.9500
C186—H186	0.9500	C98—C99	1.3900
O19—C187	1.417 (2)	C98—H98	0.9500
O19—C189	1.417 (2)	C99—C100	1.3900
C187—C188	1.517 (2)	C99—H99	0.9500
C187—H18A	0.9900	C100—H100	0.9500
C187—H18B	0.9900	C101—C102	1.384 (4)
C188—H18C	0.9800	C101—C106	1.389 (4)
C188—H18D	0.9800	C101—P4	1.838 (3)
C188—H18E	0.9800	C102—C103	1.391 (4)
C189—C190	1.518 (2)	C102—H102	0.9500
C189—H18F	0.9900	C103—C104	1.377 (5)
C189—H18G	0.9900	C103—H103	0.9500
C190—H19A	0.9800	C104—C105	1.376 (5)
C190—H19B	0.9800	C104—H104	0.9500
C190—H19C	0.9800	C105—C106	1.387 (4)
C1—O1	1.145 (4)	C105—H105	0.9500
C1—Rh1	1.914 (4)	C106—H106	0.9500
C2—C3	1.3900	C107—C108	1.385 (5)
C2—C7	1.3900	C107—C112	1.400 (4)
C2—P1	1.8483 (15)	C107—P4	1.832 (3)
C3—C4	1.3900	C108—C109	1.390 (5)
C3—H3	0.9500	C108—H108	0.9500
C4—C5	1.3900	C109—C110	1.385 (6)
C4—H4	0.9500	C109—H109	0.9500
C5—C6	1.3900	C110—C111	1.376 (6)
C5—H5	0.9500	C110—H110	0.9500
C6—C7	1.3900	C111—C112	1.378 (5)
C6—H6	0.9500	C111—H111	0.9500
C7—H7	0.9500	C112—H112	0.9500
C8—C9	1.3900	C113—O11	1.460 (3)
C8—C13	1.3900	C113—C121	1.545 (4)
C8—P1	1.8423 (16)	C113—C115	1.548 (3)
C9—C10	1.3900	C113—C114	1.608 (4)
C9—H9	0.9500	C114—O12	1.462 (3)
C10—C11	1.3900	C114—C127	1.536 (4)
C10—H10	0.9500	C114—C133	1.549 (4)
C11—C12	1.3900	C115—C116	1.3900
C11—H11	0.9500	C115—C120	1.3900
C12—C13	1.3900	C116—C117	1.3900
C12—H12	0.9500	C116—H116	0.9500
C13—H13	0.9500	C117—C118	1.3900
C14—C15	1.3900	C117—H117	0.9500
C14—C19	1.3900	C118—C119	1.3900
C14—P1	1.8514 (15)	C118—H118	0.9500
C15—C16	1.3900	C119—C120	1.3900
C15—H15	0.9500	C119—H119	0.9500
C16—C17	1.3900	C120—H120	0.9500
C16—H16	0.9500	C121—C122	1.384 (4)
C17—C18	1.3900	C121—C126	1.398 (4)
C17—H17	0.9500	C122—C123	1.395 (4)
C18—C19	1.3900	C122—H122	0.9500
C18—H18	0.9500	C123—C124	1.373 (5)
C19—H19	0.9500	C123—H123	0.9500
C20—O2	1.461 (3)	C124—C125	1.370 (5)
C20—C28	1.539 (3)	C124—H124	0.9500
C20—C22	1.540 (4)	C125—C126	1.394 (4)
C20—C21	1.619 (4)	C125—H125	0.9500
C21—O3	1.456 (3)	C126—H126	0.9500
C21—C40	1.530 (4)	C127—C128	1.383 (5)
C21—C34	1.548 (4)	C127—C132	1.387 (5)
C22—C27	1.381 (4)	C128—C129	1.403 (5)
C22—C23	1.395 (4)	C128—H128	0.9500
C23—C24	1.389 (4)	C129—C130	1.382 (7)
C23—H23	0.9500	C129—H129	0.9500
C24—C25	1.387 (4)	C130—C131	1.380 (7)
C24—H24	0.9500	C130—H130	0.9500
C25—C26	1.373 (5)	C131—C132	1.392 (5)
C25—H25	0.9500	C131—H131	0.9500
C26—C27	1.395 (4)	C132—H132	0.9500
C26—H26	0.9500	C133—C138	1.381 (4)
C27—H27	0.9500	C133—C134	1.391 (4)
C28—C29	1.3900	C134—C135	1.389 (4)
C28—C33	1.3900	C134—H134	0.9500
C29—C30	1.3900	C135—C136	1.376 (4)
C29—H29	0.9500	C135—H135	0.9500
C30—C31	1.3900	C136—C137	1.380 (5)
C30—H30	0.9500	C136—H136	0.9500
C31—C32	1.3900	C137—C138	1.389 (4)
C31—H31	0.9500	C137—H137	0.9500
C32—C33	1.3900	C138—H138	0.9500
C32—H32	0.9500	C139—O13	1.393 (3)
C33—H33	0.9500	C139—C144	1.395 (4)
C34—C35	1.392 (4)	C139—C140	1.409 (4)
C34—C39	1.394 (4)	C140—C141	1.408 (4)
C35—C36	1.393 (4)	C140—C151	1.543 (5)
C35—H35	0.9500	C141—C142	1.368 (5)
C36—C37	1.384 (5)	C141—H141	0.9500
C36—H36	0.9500	C142—O14	1.367 (3)
C37—C38	1.375 (5)	C142—C143	1.386 (4)
C37—H37	0.9500	C143—C144	1.394 (4)
C38—C39	1.389 (4)	C143—H143	0.9500
C38—H38	0.9500	C144—C145	1.494 (4)
C39—H39	0.9500	C145—C146	1.386 (4)
C40—C45	1.386 (4)	C145—C150	1.409 (4)
C40—C41	1.398 (4)	C146—C147	1.394 (4)
C41—C42	1.381 (5)	C146—H146	0.9500
C41—H41	0.9500	C147—O15	1.367 (3)
C42—C43	1.381 (5)	C147—C148	1.384 (4)
C42—H42	0.9500	C148—C149	1.407 (4)
C43—C44	1.387 (5)	C148—H148	0.9500
C43—H43	0.9500	C149—C150	1.405 (4)
C44—C45	1.388 (4)	C149—C157	1.541 (4)
C44—H44	0.9500	C150—O18	1.390 (3)
C45—H45	0.9500	C151—C154	1.527 (5)
C46—C51	1.386 (4)	C151—C152	1.528 (5)
C46—O4	1.404 (3)	C151—C153	1.544 (4)
C46—C47	1.410 (4)	C152—H15A	0.9800
C47—C48	1.395 (4)	C152—H15B	0.9800
C47—C58	1.533 (5)	C152—H15C	0.9800
C48—C49	1.368 (5)	C153—H15D	0.9800
C48—H48	0.9500	C153—H15E	0.9800
C49—C50	1.378 (4)	C153—H15F	0.9800
C49—O5	1.388 (3)	C154—H15G	0.9800
C50—C51	1.406 (4)	C154—H15H	0.9800
C50—H50	0.9500	C154—H15I	0.9800
C51—C52	1.498 (4)	C155—O14	1.417 (4)
C52—C57	1.395 (4)	C155—H15J	0.9800
C52—C53	1.398 (4)	C155—H15K	0.9800
C53—C54	1.378 (4)	C155—H15L	0.9800
C53—H53	0.9500	C156—O15	1.425 (4)
C54—O6	1.382 (3)	C156—H15M	0.9800
C54—C55	1.393 (4)	C156—H15N	0.9800
C55—C56	1.393 (4)	C156—H15O	0.9800
C55—H55	0.9500	C157—C160	1.525 (4)
C56—C57	1.405 (4)	C157—C158	1.536 (4)
C56—C64	1.533 (4)	C157—C159	1.542 (4)
C57—O9	1.395 (3)	C158—H15P	0.9800
C58—C61	1.532 (4)	C158—H15Q	0.9800
C58—C60	1.535 (5)	C158—H15R	0.9800
C58—C59	1.544 (5)	C159—H15S	0.9800
C59—H59A	0.9800	C159—H15T	0.9800
C59—H59B	0.9800	C159—H15U	0.9800
C59—H59C	0.9800	C160—H16A	0.9800
C60—H60A	0.9800	C160—H16B	0.9800
C60—H60B	0.9800	C160—H16C	0.9800
C60—H60C	0.9800	C161—O16	1.455 (3)
C61—H61A	0.9800	C161—C163	1.530 (4)
C61—H61B	0.9800	C161—C169	1.542 (4)
C61—H61C	0.9800	C161—C162	1.642 (4)
C62—O5	1.413 (5)	C162—O17	1.456 (3)
C62—H62A	0.9800	C162—C175	1.548 (4)
C62—H62B	0.9800	C163—C168	1.382 (4)
C62—H62C	0.9800	C163—C164	1.399 (4)
C63—O6	1.440 (4)	C164—C165	1.383 (5)
C63—H63A	0.9800	C164—H164	0.9500
C63—H63B	0.9800	C165—C166	1.379 (5)
C63—H63C	0.9800	C165—H165	0.9500
C64—C65	1.530 (5)	C166—C167	1.387 (5)
C64—C66	1.537 (4)	C166—H166	0.9500
C64—C67	1.538 (4)	C167—C168	1.384 (5)
C65—H65A	0.9800	C167—H167	0.9500
C65—H65B	0.9800	C168—H168	0.9500
C65—H65C	0.9800	C169—C174	1.390 (4)
C66—H66A	0.9800	C169—C170	1.391 (5)
C66—H66B	0.9800	C170—C171	1.388 (5)
C66—H66C	0.9800	C170—H170	0.9500
C67—H67A	0.9800	C171—C172	1.378 (5)
C67—H67B	0.9800	C171—H171	0.9500
C67—H67C	0.9800	C172—C173	1.379 (5)
C68—O7	1.456 (3)	C172—H172	0.9500
C68—C70	1.529 (3)	C173—C174	1.391 (5)
C68—C76	1.560 (3)	C173—H173	0.9500
C68—C69	1.631 (4)	C174—H174	0.9500
C69—O8	1.462 (3)	C175—C176	1.3900
C69—C88	1.525 (4)	C175—C180	1.3900
C69—C82	1.553 (3)	C176—C177	1.3900
C70—C71	1.3900	C176—H176	0.9500
C70—C75	1.3900	C177—C178	1.3900
C71—C72	1.3900	C177—H177	0.9500
C71—H71	0.9500	C178—C179	1.3900
C72—C73	1.3900	C178—H178	0.9500
C72—H72	0.9500	C179—C180	1.3900
C73—C74	1.3900	C179—H179	0.9500
C73—H73	0.9500	C180—H180	0.9500
C74—C75	1.3900	O2—P2	1.622 (2)
C74—H74	0.9500	O3—P2	1.6135 (19)
C75—H75	0.9500	O4—P2	1.6412 (18)
C76—C77	1.3900	O7—P3	1.6150 (19)
C76—C81	1.3900	O8—P3	1.6117 (19)
C77—C78	1.3900	O9—P3	1.639 (2)
C77—H77	0.9500	O11—P6	1.6130 (19)
C78—C79	1.3900	O12—P6	1.626 (2)
C78—H78	0.9500	O13—P6	1.6419 (18)
C79—C80	1.3900	O16—P5	1.6148 (18)
C79—H79	0.9500	O17—P5	1.6199 (18)
C80—C81	1.3900	O18—P5	1.646 (2)
C80—H80	0.9500	P1—Rh1	2.3221 (7)
C81—H81	0.9500	P2—Rh1	2.2447 (7)
C82—C83	1.3900	P3—Rh1	2.2569 (7)
C82—C87	1.3900	P4—Rh2	2.3209 (7)
C83—C84	1.3900	P5—Rh2	2.2555 (7)
C83—H83	0.9500	P6—Rh2	2.2553 (7)
C84—C85	1.3900	Rh1—H1	1.40 (4)
C84—H84	0.9500	Rh2—H2	1.40 (4)
C85—C86	1.3900	O20—C191	1.429 (8)
C85—H85	0.9500	O20—C193	1.433 (8)
C86—C87	1.3900	C192—C191	1.517 (2)
C86—H86	0.9500	C192—H19D	0.9800
C87—H87	0.9500	C192—H19E	0.9800
C88—C93	1.382 (5)	C192—H19F	0.9800
C88—C89	1.403 (4)	C191—H19G	0.9900
C89—C90	1.384 (5)	C191—H19H	0.9900
C89—H89	0.9500	C193—C194	1.519 (2)
C90—C91	1.380 (6)	C193—H19I	0.9900
C90—H90	0.9500	C193—H19J	0.9900
C91—C92	1.378 (6)	C194—H19K	0.9800
C91—H91	0.9500	C194—H19L	0.9800
C92—C93	1.379 (5)	C194—H19M	0.9800
			
C182—C181—C186	120.0	C95—C100—H100	120.0
C182—C181—C162	121.45 (18)	C102—C101—C106	118.9 (3)
C186—C181—C162	118.41 (18)	C102—C101—P4	124.1 (2)
C183—C182—C181	120.0	C106—C101—P4	116.9 (2)
C183—C182—H182	120.0	C101—C102—C103	120.1 (3)
C181—C182—H182	120.0	C101—C102—H102	119.9
C184—C183—C182	120.0	C103—C102—H102	119.9
C184—C183—H183	120.0	C104—C103—C102	120.6 (3)
C182—C183—H183	120.0	C104—C103—H103	119.7
C183—C184—C185	120.0	C102—C103—H103	119.7
C183—C184—H184	120.0	C105—C104—C103	119.5 (3)
C185—C184—H184	120.0	C105—C104—H104	120.3
C186—C185—C184	120.0	C103—C104—H104	120.3
C186—C185—H185	120.0	C104—C105—C106	120.3 (3)
C184—C185—H185	120.0	C104—C105—H105	119.8
C185—C186—C181	120.0	C106—C105—H105	119.8
C185—C186—H186	120.0	C105—C106—C101	120.5 (3)
C181—C186—H186	120.0	C105—C106—H106	119.7
C187—O19—C189	107.0 (7)	C101—C106—H106	119.7
O19—C187—C188	105.6 (6)	C108—C107—C112	118.9 (3)
O19—C187—H18A	110.6	C108—C107—P4	119.1 (2)
C188—C187—H18A	110.6	C112—C107—P4	122.0 (3)
O19—C187—H18B	110.6	C107—C108—C109	120.7 (4)
C188—C187—H18B	110.6	C107—C108—H108	119.6
H18A—C187—H18B	108.7	C109—C108—H108	119.6
C187—C188—H18C	109.5	C110—C109—C108	119.3 (4)
C187—C188—H18D	109.5	C110—C109—H109	120.3
H18C—C188—H18D	109.5	C108—C109—H109	120.3
C187—C188—H18E	109.5	C111—C110—C109	120.6 (4)
H18C—C188—H18E	109.5	C111—C110—H110	119.7
H18D—C188—H18E	109.5	C109—C110—H110	119.7
O19—C189—C190	102.6 (7)	C110—C111—C112	120.1 (4)
O19—C189—H18F	111.2	C110—C111—H111	120.0
C190—C189—H18F	111.2	C112—C111—H111	120.0
O19—C189—H18G	111.2	C111—C112—C107	120.4 (4)
C190—C189—H18G	111.2	C111—C112—H112	119.8
H18F—C189—H18G	109.2	C107—C112—H112	119.8
C189—C190—H19A	109.5	O11—C113—C121	108.1 (2)
C189—C190—H19B	109.5	O11—C113—C115	106.26 (19)
H19A—C190—H19B	109.5	C121—C113—C115	109.3 (2)
C189—C190—H19C	109.5	O11—C113—C114	103.1 (2)
H19A—C190—H19C	109.5	C121—C113—C114	116.5 (2)
H19B—C190—H19C	109.5	C115—C113—C114	112.9 (2)
O1—C1—Rh1	178.3 (3)	O12—C114—C127	106.9 (2)
C3—C2—C7	120.0	O12—C114—C133	105.9 (2)
C3—C2—P1	117.11 (11)	C127—C114—C133	109.9 (2)
C7—C2—P1	122.83 (11)	O12—C114—C113	105.0 (2)
C4—C3—C2	120.0	C127—C114—C113	116.9 (2)
C4—C3—H3	120.0	C133—C114—C113	111.5 (2)
C2—C3—H3	120.0	C116—C115—C120	120.0
C3—C4—C5	120.0	C116—C115—C113	119.75 (14)
C3—C4—H4	120.0	C120—C115—C113	119.99 (14)
C5—C4—H4	120.0	C115—C116—C117	120.0
C6—C5—C4	120.0	C115—C116—H116	120.0
C6—C5—H5	120.0	C117—C116—H116	120.0
C4—C5—H5	120.0	C118—C117—C116	120.0
C5—C6—C7	120.0	C118—C117—H117	120.0
C5—C6—H6	120.0	C116—C117—H117	120.0
C7—C6—H6	120.0	C119—C118—C117	120.0
C6—C7—C2	120.0	C119—C118—H118	120.0
C6—C7—H7	120.0	C117—C118—H118	120.0
C2—C7—H7	120.0	C118—C119—C120	120.0
C9—C8—C13	120.0	C118—C119—H119	120.0
C9—C8—P1	121.94 (11)	C120—C119—H119	120.0
C13—C8—P1	118.05 (11)	C119—C120—C115	120.0
C8—C9—C10	120.0	C119—C120—H120	120.0
C8—C9—H9	120.0	C115—C120—H120	120.0
C10—C9—H9	120.0	C122—C121—C126	117.8 (3)
C11—C10—C9	120.0	C122—C121—C113	120.7 (3)
C11—C10—H10	120.0	C126—C121—C113	121.5 (3)
C9—C10—H10	120.0	C121—C122—C123	121.1 (3)
C10—C11—C12	120.0	C121—C122—H122	119.4
C10—C11—H11	120.0	C123—C122—H122	119.4
C12—C11—H11	120.0	C124—C123—C122	120.3 (3)
C11—C12—C13	120.0	C124—C123—H123	119.9
C11—C12—H12	120.0	C122—C123—H123	119.9
C13—C12—H12	120.0	C125—C124—C123	119.6 (3)
C12—C13—C8	120.0	C125—C124—H124	120.2
C12—C13—H13	120.0	C123—C124—H124	120.2
C8—C13—H13	120.0	C124—C125—C126	120.6 (3)
C15—C14—C19	120.0	C124—C125—H125	119.7
C15—C14—P1	120.13 (11)	C126—C125—H125	119.7
C19—C14—P1	118.69 (11)	C125—C126—C121	120.6 (3)
C14—C15—C16	120.0	C125—C126—H126	119.7
C14—C15—H15	120.0	C121—C126—H126	119.7
C16—C15—H15	120.0	C128—C127—C132	118.5 (3)
C17—C16—C15	120.0	C128—C127—C114	118.2 (3)
C17—C16—H16	120.0	C132—C127—C114	123.1 (3)
C15—C16—H16	120.0	C127—C128—C129	120.6 (4)
C16—C17—C18	120.0	C127—C128—H128	119.7
C16—C17—H17	120.0	C129—C128—H128	119.7
C18—C17—H17	120.0	C130—C129—C128	120.2 (5)
C17—C18—C19	120.0	C130—C129—H129	119.9
C17—C18—H18	120.0	C128—C129—H129	119.9
C19—C18—H18	120.0	C131—C130—C129	119.6 (4)
C18—C19—C14	120.0	C131—C130—H130	120.2
C18—C19—H19	120.0	C129—C130—H130	120.2
C14—C19—H19	120.0	C130—C131—C132	120.0 (4)
O2—C20—C28	107.8 (2)	C130—C131—H131	120.0
O2—C20—C22	104.6 (2)	C132—C131—H131	120.0
C28—C20—C22	110.0 (2)	C127—C132—C131	121.2 (4)
O2—C20—C21	104.92 (19)	C127—C132—H132	119.4
C28—C20—C21	114.3 (2)	C131—C132—H132	119.4
C22—C20—C21	114.4 (2)	C138—C133—C134	118.2 (3)
O3—C21—C40	105.8 (2)	C138—C133—C114	122.1 (3)
O3—C21—C34	107.8 (2)	C134—C133—C114	119.7 (2)
C40—C21—C34	107.5 (2)	C135—C134—C133	120.9 (3)
O3—C21—C20	104.0 (2)	C135—C134—H134	119.6
C40—C21—C20	117.2 (2)	C133—C134—H134	119.6
C34—C21—C20	113.8 (2)	C136—C135—C134	120.3 (3)
C27—C22—C23	118.3 (3)	C136—C135—H135	119.9
C27—C22—C20	122.6 (3)	C134—C135—H135	119.9
C23—C22—C20	119.1 (2)	C135—C136—C137	119.4 (3)
C24—C23—C22	120.8 (3)	C135—C136—H136	120.3
C24—C23—H23	119.6	C137—C136—H136	120.3
C22—C23—H23	119.6	C136—C137—C138	120.4 (3)
C25—C24—C23	120.1 (3)	C136—C137—H137	119.8
C25—C24—H24	119.9	C138—C137—H137	119.8
C23—C24—H24	119.9	C133—C138—C137	121.0 (3)
C26—C25—C24	119.5 (3)	C133—C138—H138	119.5
C26—C25—H25	120.2	C137—C138—H138	119.5
C24—C25—H25	120.2	O13—C139—C144	120.6 (2)
C25—C26—C27	120.3 (3)	O13—C139—C140	117.9 (3)
C25—C26—H26	119.8	C144—C139—C140	121.4 (2)
C27—C26—H26	119.8	C141—C140—C139	116.6 (3)
C22—C27—C26	121.0 (3)	C141—C140—C151	119.5 (3)
C22—C27—H27	119.5	C139—C140—C151	123.9 (3)
C26—C27—H27	119.5	C142—C141—C140	122.4 (3)
C29—C28—C33	120.0	C142—C141—H141	118.8
C29—C28—C20	119.83 (16)	C140—C141—H141	118.8
C33—C28—C20	120.10 (16)	O14—C142—C141	124.9 (3)
C30—C29—C28	120.0	O14—C142—C143	115.0 (3)
C30—C29—H29	120.0	C141—C142—C143	120.0 (3)
C28—C29—H29	120.0	C142—C143—C144	119.9 (3)
C29—C30—C31	120.0	C142—C143—H143	120.0
C29—C30—H30	120.0	C144—C143—H143	120.0
C31—C30—H30	120.0	C143—C144—C139	119.5 (2)
C30—C31—C32	120.0	C143—C144—C145	116.0 (3)
C30—C31—H31	120.0	C139—C144—C145	124.4 (2)
C32—C31—H31	120.0	C146—C145—C150	119.3 (2)
C31—C32—C33	120.0	C146—C145—C144	119.7 (2)
C31—C32—H32	120.0	C150—C145—C144	120.9 (2)
C33—C32—H32	120.0	C145—C146—C147	119.9 (2)
C32—C33—C28	120.0	C145—C146—H146	120.0
C32—C33—H33	120.0	C147—C146—H146	120.0
C28—C33—H33	120.0	O15—C147—C148	124.9 (3)
C35—C34—C39	117.6 (3)	O15—C147—C146	115.0 (2)
C35—C34—C21	120.5 (2)	C148—C147—C146	120.1 (2)
C39—C34—C21	121.9 (3)	C147—C148—C149	121.9 (3)
C34—C35—C36	120.9 (3)	C147—C148—H148	119.1
C34—C35—H35	119.6	C149—C148—H148	119.1
C36—C35—H35	119.6	C150—C149—C148	116.7 (2)
C37—C36—C35	120.3 (3)	C150—C149—C157	122.7 (2)
C37—C36—H36	119.8	C148—C149—C157	120.5 (3)
C35—C36—H36	119.8	O18—C150—C149	119.3 (2)
C38—C37—C36	119.6 (3)	O18—C150—C145	118.9 (2)
C38—C37—H37	120.2	C149—C150—C145	121.8 (2)
C36—C37—H37	120.2	C154—C151—C152	110.2 (3)
C37—C38—C39	120.0 (3)	C154—C151—C140	110.7 (3)
C37—C38—H38	120.0	C152—C151—C140	111.1 (3)
C39—C38—H38	120.0	C154—C151—C153	107.4 (3)
C38—C39—C34	121.5 (3)	C152—C151—C153	105.9 (3)
C38—C39—H39	119.2	C140—C151—C153	111.3 (3)
C34—C39—H39	119.2	C151—C152—H15A	109.5
C45—C40—C41	118.1 (3)	C151—C152—H15B	109.5
C45—C40—C21	118.1 (2)	H15A—C152—H15B	109.5
C41—C40—C21	123.1 (3)	C151—C152—H15C	109.5
C42—C41—C40	120.7 (3)	H15A—C152—H15C	109.5
C42—C41—H41	119.6	H15B—C152—H15C	109.5
C40—C41—H41	119.6	C151—C153—H15D	109.5
C41—C42—C43	120.6 (3)	C151—C153—H15E	109.5
C41—C42—H42	119.7	H15D—C153—H15E	109.5
C43—C42—H42	119.7	C151—C153—H15F	109.5
C42—C43—C44	119.3 (3)	H15D—C153—H15F	109.5
C42—C43—H43	120.3	H15E—C153—H15F	109.5
C44—C43—H43	120.3	C151—C154—H15G	109.5
C43—C44—C45	120.0 (3)	C151—C154—H15H	109.5
C43—C44—H44	120.0	H15G—C154—H15H	109.5
C45—C44—H44	120.0	C151—C154—H15I	109.5
C40—C45—C44	121.2 (3)	H15G—C154—H15I	109.5
C40—C45—H45	119.4	H15H—C154—H15I	109.5
C44—C45—H45	119.4	O14—C155—H15J	109.5
C51—C46—O4	120.6 (2)	O14—C155—H15K	109.5
C51—C46—C47	121.7 (2)	H15J—C155—H15K	109.5
O4—C46—C47	117.3 (3)	O14—C155—H15L	109.5
C48—C47—C46	116.3 (3)	H15J—C155—H15L	109.5
C48—C47—C58	119.6 (3)	H15K—C155—H15L	109.5
C46—C47—C58	123.9 (3)	O15—C156—H15M	109.5
C49—C48—C47	122.6 (3)	O15—C156—H15N	109.5
C49—C48—H48	118.7	H15M—C156—H15N	109.5
C47—C48—H48	118.7	O15—C156—H15O	109.5
C48—C49—C50	120.6 (3)	H15M—C156—H15O	109.5
C48—C49—O5	115.9 (3)	H15N—C156—H15O	109.5
C50—C49—O5	123.4 (3)	C160—C157—C158	106.3 (2)
C49—C50—C51	119.1 (3)	C160—C157—C149	112.2 (3)
C49—C50—H50	120.5	C158—C157—C149	111.9 (2)
C51—C50—H50	120.5	C160—C157—C159	110.1 (3)
C46—C51—C50	119.5 (3)	C158—C157—C159	107.4 (3)
C46—C51—C52	125.2 (2)	C149—C157—C159	108.8 (2)
C50—C51—C52	115.1 (3)	C157—C158—H15P	109.5
C57—C52—C53	119.6 (3)	C157—C158—H15Q	109.5
C57—C52—C51	122.0 (2)	H15P—C158—H15Q	109.5
C53—C52—C51	118.1 (2)	C157—C158—H15R	109.5
C54—C53—C52	119.0 (3)	H15P—C158—H15R	109.5
C54—C53—H53	120.5	H15Q—C158—H15R	109.5
C52—C53—H53	120.5	C157—C159—H15S	109.5
C53—C54—O6	124.4 (3)	C157—C159—H15T	109.5
C53—C54—C55	121.0 (3)	H15S—C159—H15T	109.5
O6—C54—C55	114.6 (3)	C157—C159—H15U	109.5
C56—C55—C54	121.7 (3)	H15S—C159—H15U	109.5
C56—C55—H55	119.1	H15T—C159—H15U	109.5
C54—C55—H55	119.1	C157—C160—H16A	109.5
C55—C56—C57	116.5 (3)	C157—C160—H16B	109.5
C55—C56—C64	119.7 (3)	H16A—C160—H16B	109.5
C57—C56—C64	123.7 (2)	C157—C160—H16C	109.5
O9—C57—C52	119.2 (2)	H16A—C160—H16C	109.5
O9—C57—C56	118.6 (2)	H16B—C160—H16C	109.5
C52—C57—C56	122.2 (2)	O16—C161—C163	106.1 (2)
C61—C58—C47	111.6 (3)	O16—C161—C169	108.0 (2)
C61—C58—C60	110.9 (3)	C163—C161—C169	110.7 (2)
C47—C58—C60	109.4 (3)	O16—C161—C162	103.63 (19)
C61—C58—C59	105.8 (3)	C163—C161—C162	113.4 (2)
C47—C58—C59	111.6 (3)	C169—C161—C162	114.2 (2)
C60—C58—C59	107.4 (3)	O17—C162—C181	107.8 (2)
C58—C59—H59A	109.5	O17—C162—C175	105.6 (2)
C58—C59—H59B	109.5	C181—C162—C175	110.2 (2)
H59A—C59—H59B	109.5	O17—C162—C161	104.14 (19)
C58—C59—H59C	109.5	C181—C162—C161	116.1 (2)
H59A—C59—H59C	109.5	C175—C162—C161	112.2 (2)
H59B—C59—H59C	109.5	C168—C163—C164	118.3 (3)
C58—C60—H60A	109.5	C168—C163—C161	120.5 (3)
C58—C60—H60B	109.5	C164—C163—C161	121.3 (3)
H60A—C60—H60B	109.5	C165—C164—C163	120.7 (3)
C58—C60—H60C	109.5	C165—C164—H164	119.6
H60A—C60—H60C	109.5	C163—C164—H164	119.6
H60B—C60—H60C	109.5	C166—C165—C164	120.7 (3)
C58—C61—H61A	109.5	C166—C165—H165	119.7
C58—C61—H61B	109.5	C164—C165—H165	119.7
H61A—C61—H61B	109.5	C165—C166—C167	118.7 (3)
C58—C61—H61C	109.5	C165—C166—H166	120.6
H61A—C61—H61C	109.5	C167—C166—H166	120.6
H61B—C61—H61C	109.5	C168—C167—C166	120.9 (4)
O5—C62—H62A	109.5	C168—C167—H167	119.6
O5—C62—H62B	109.5	C166—C167—H167	119.6
H62A—C62—H62B	109.5	C163—C168—C167	120.7 (3)
O5—C62—H62C	109.5	C163—C168—H168	119.7
H62A—C62—H62C	109.5	C167—C168—H168	119.7
H62B—C62—H62C	109.5	C174—C169—C170	117.7 (3)
O6—C63—H63A	109.5	C174—C169—C161	121.6 (3)
O6—C63—H63B	109.5	C170—C169—C161	120.7 (3)
H63A—C63—H63B	109.5	C171—C170—C169	121.2 (3)
O6—C63—H63C	109.5	C171—C170—H170	119.4
H63A—C63—H63C	109.5	C169—C170—H170	119.4
H63B—C63—H63C	109.5	C172—C171—C170	120.6 (4)
C65—C64—C56	111.8 (2)	C172—C171—H171	119.7
C65—C64—C66	107.0 (3)	C170—C171—H171	119.7
C56—C64—C66	109.6 (2)	C171—C172—C173	118.9 (3)
C65—C64—C67	107.3 (3)	C171—C172—H172	120.6
C56—C64—C67	111.7 (3)	C173—C172—H172	120.6
C66—C64—C67	109.3 (3)	C172—C173—C174	120.8 (3)
C64—C65—H65A	109.5	C172—C173—H173	119.6
C64—C65—H65B	109.5	C174—C173—H173	119.6
H65A—C65—H65B	109.5	C169—C174—C173	120.8 (3)
C64—C65—H65C	109.5	C169—C174—H174	119.6
H65A—C65—H65C	109.5	C173—C174—H174	119.6
H65B—C65—H65C	109.5	C176—C175—C180	120.0
C64—C66—H66A	109.5	C176—C175—C162	118.47 (17)
C64—C66—H66B	109.5	C180—C175—C162	121.52 (17)
H66A—C66—H66B	109.5	C177—C176—C175	120.0
C64—C66—H66C	109.5	C177—C176—H176	120.0
H66A—C66—H66C	109.5	C175—C176—H176	120.0
H66B—C66—H66C	109.5	C176—C177—C178	120.0
C64—C67—H67A	109.5	C176—C177—H177	120.0
C64—C67—H67B	109.5	C178—C177—H177	120.0
H67A—C67—H67B	109.5	C177—C178—C179	120.0
C64—C67—H67C	109.5	C177—C178—H178	120.0
H67A—C67—H67C	109.5	C179—C178—H178	120.0
H67B—C67—H67C	109.5	C180—C179—C178	120.0
O7—C68—C70	106.3 (2)	C180—C179—H179	120.0
O7—C68—C76	106.3 (2)	C178—C179—H179	120.0
C70—C68—C76	108.60 (19)	C179—C180—C175	120.0
O7—C68—C69	104.31 (19)	C179—C180—H180	120.0
C70—C68—C69	118.5 (2)	C175—C180—H180	120.0
C76—C68—C69	111.9 (2)	C20—O2—P2	117.82 (17)
O8—C69—C88	105.9 (2)	C21—O3—P2	118.67 (17)
O8—C69—C82	108.01 (19)	C46—O4—P2	129.59 (17)
C88—C69—C82	111.3 (2)	C49—O5—C62	116.8 (3)
O8—C69—C68	102.87 (19)	C54—O6—C63	115.6 (3)
C88—C69—C68	113.1 (2)	C68—O7—P3	118.27 (16)
C82—C69—C68	114.8 (2)	C69—O8—P3	118.24 (17)
C71—C70—C75	120.0	C57—O9—P3	124.14 (17)
C71—C70—C68	116.89 (15)	C113—O11—P6	117.13 (17)
C75—C70—C68	122.57 (16)	C114—O12—P6	116.84 (18)
C70—C71—C72	120.0	C139—O13—P6	127.78 (16)
C70—C71—H71	120.0	C142—O14—C155	117.0 (3)
C72—C71—H71	120.0	C147—O15—C156	117.2 (2)
C73—C72—C71	120.0	C161—O16—P5	118.77 (16)
C73—C72—H72	120.0	C162—O17—P5	118.79 (15)
C71—C72—H72	120.0	C150—O18—P5	122.75 (17)
C72—C73—C74	120.0	C8—P1—C2	102.39 (9)
C72—C73—H73	120.0	C8—P1—C14	102.36 (9)
C74—C73—H73	120.0	C2—P1—C14	99.09 (9)
C75—C74—C73	120.0	C8—P1—Rh1	116.38 (7)
C75—C74—H74	120.0	C2—P1—Rh1	114.35 (7)
C73—C74—H74	120.0	C14—P1—Rh1	119.52 (7)
C74—C75—C70	120.0	O3—P2—O2	93.57 (10)
C74—C75—H75	120.0	O3—P2—O4	102.08 (10)
C70—C75—H75	120.0	O2—P2—O4	97.60 (10)
C77—C76—C81	120.0	O3—P2—Rh1	122.83 (7)
C77—C76—C68	121.55 (16)	O2—P2—Rh1	120.72 (7)
C81—C76—C68	118.44 (16)	O4—P2—Rh1	115.15 (7)
C76—C77—C78	120.0	O8—P3—O7	93.33 (9)
C76—C77—H77	120.0	O8—P3—O9	102.00 (10)
C78—C77—H77	120.0	O7—P3—O9	98.97 (11)
C79—C78—C77	120.0	O8—P3—Rh1	123.67 (8)
C79—C78—H78	120.0	O7—P3—Rh1	118.87 (7)
C77—C78—H78	120.0	O9—P3—Rh1	115.28 (7)
C80—C79—C78	120.0	C107—P4—C101	102.62 (14)
C80—C79—H79	120.0	C107—P4—C95	102.58 (11)
C78—C79—H79	120.0	C101—P4—C95	99.81 (11)
C79—C80—C81	120.0	C107—P4—Rh2	116.69 (10)
C79—C80—H80	120.0	C101—P4—Rh2	113.21 (9)
C81—C80—H80	120.0	C95—P4—Rh2	119.36 (6)
C80—C81—C76	120.0	O16—P5—O17	93.19 (9)
C80—C81—H81	120.0	O16—P5—O18	102.91 (10)
C76—C81—H81	120.0	O17—P5—O18	98.55 (11)
C83—C82—C87	120.0	O16—P5—Rh2	122.23 (8)
C83—C82—C69	119.75 (16)	O17—P5—Rh2	120.32 (8)
C87—C82—C69	120.22 (16)	O18—P5—Rh2	115.10 (7)
C84—C83—C82	120.0	O11—P6—O12	93.41 (10)
C84—C83—H83	120.0	O11—P6—O13	103.04 (10)
C82—C83—H83	120.0	O12—P6—O13	97.12 (10)
C85—C84—C83	120.0	O11—P6—Rh2	120.49 (7)
C85—C84—H84	120.0	O12—P6—Rh2	122.85 (8)
C83—C84—H84	120.0	O13—P6—Rh2	115.26 (7)
C84—C85—C86	120.0	C1—Rh1—P2	99.50 (9)
C84—C85—H85	120.0	C1—Rh1—P3	99.19 (9)
C86—C85—H85	120.0	P2—Rh1—P3	117.18 (2)
C85—C86—C87	120.0	C1—Rh1—P1	94.90 (9)
C85—C86—H86	120.0	P2—Rh1—P1	120.36 (2)
C87—C86—H86	120.0	P3—Rh1—P1	116.96 (3)
C86—C87—C82	120.0	C1—Rh1—H1	173.7 (16)
C86—C87—H87	120.0	P2—Rh1—H1	86.8 (15)
C82—C87—H87	120.0	P3—Rh1—H1	78.3 (15)
C93—C88—C89	117.8 (3)	P1—Rh1—H1	81.2 (15)
C93—C88—C69	121.1 (3)	C94—Rh2—P6	101.63 (9)
C89—C88—C69	121.0 (3)	C94—Rh2—P5	98.77 (10)
C90—C89—C88	120.5 (3)	P6—Rh2—P5	117.99 (2)
C90—C89—H89	119.7	C94—Rh2—P4	95.09 (9)
C88—C89—H89	119.7	P6—Rh2—P4	118.93 (2)
C91—C90—C89	120.5 (3)	P5—Rh2—P4	116.62 (3)
C91—C90—H90	119.7	C94—Rh2—H2	176.3 (15)
C89—C90—H90	119.7	P6—Rh2—H2	82.1 (15)
C92—C91—C90	119.1 (4)	P5—Rh2—H2	79.1 (15)
C92—C91—H91	120.4	P4—Rh2—H2	83.2 (15)
C90—C91—H91	120.4	C191—O20—C193	105.9 (7)
C91—C92—C93	120.6 (4)	C191—C192—H19D	109.5
C91—C92—H92	119.7	C191—C192—H19E	109.5
C93—C92—H92	119.7	H19D—C192—H19E	109.5
C92—C93—C88	121.3 (3)	C191—C192—H19F	109.5
C92—C93—H93	119.4	H19D—C192—H19F	109.5
C88—C93—H93	119.4	H19E—C192—H19F	109.5
O10—C94—Rh2	178.2 (3)	O20—C191—C192	104.1 (8)
C96—C95—C100	120.0	O20—C191—H19G	110.9
C96—C95—P4	121.00 (10)	C192—C191—H19G	110.9
C100—C95—P4	118.11 (10)	O20—C191—H19H	110.9
C95—C96—C97	120.0	C192—C191—H19H	110.9
C95—C96—H96	120.0	H19G—C191—H19H	109.0
C97—C96—H96	120.0	O20—C193—C194	106.7 (10)
C98—C97—C96	120.0	O20—C193—H19I	110.4
C98—C97—H97	120.0	C194—C193—H19I	110.4
C96—C97—H97	120.0	O20—C193—H19J	110.4
C97—C98—C99	120.0	C194—C193—H19J	110.4
C97—C98—H98	120.0	H19I—C193—H19J	108.6
C99—C98—H98	120.0	C193—C194—H19K	109.5
C100—C99—C98	120.0	C193—C194—H19L	109.5
C100—C99—H99	120.0	H19K—C194—H19L	109.5
C98—C99—H99	120.0	C193—C194—H19M	109.5
C99—C100—C95	120.0	H19K—C194—H19M	109.5
C99—C100—H100	120.0	H19L—C194—H19M	109.5
